# Characterization of *SPP1* + macrophages and their correlation with resistance to neoadjuvant chemotherapy in colorectal cancer liver metastasis

**DOI:** 10.1186/s43556-026-00581-7

**Published:** 2026-10-05

**Authors:** Jingzhi Tang, Weijie Hao, Xigan He, Xin Qian, Linjie Ruan, Mengyao Quan, Jian Zhou, Jing Tang, Lu Wang, Yi Guo, Yiming Zhao, Cai Chang, Shichong Zhou

**Affiliations:** 1 Department of Ultrasonography, Fudan University Shanghai Cancer Center, Shanghai, 200032 China; 2 Department of Oncology, Shanghai Medical College, Fudan University, Shanghai, 200032 China; 3 State Key Laboratory of Oncogenes and Related Genes, Renji-Med X Clinical Stem Cell Research Center, Ren Ji Hospital, School of Medicine, Shanghai Jiao Tong University, Shanghai, 200032 China; 4 Department of Hepatic Surgery, Fudan University Shanghai Cancer Center, Shanghai, 200032 China; 5 Department of Vascular Biology, European Center for Angioscience (ECAS), Medical Faculty Mannheim, Heidelberg University, 68167 Mannheim, Germany; 6 Department of Integrative Oncology, Fudan University Shanghai Cancer Center, Shanghai, 200032 China; 7 Research Program in Systems Oncology, Faculty of Medicine, University of Helsinki, 00290 Helsinki, Finland; 8 Department of Electronic Engineering, School of Information Science and Technology, Fudan University, Shanghai, 200433 China; 9 The Key Laboratory of Medical Imaging Computing and Computer Assisted Intervention (MICCAI) of Shanghai, Shanghai, 200032 China

**Keywords:** Colorectal cancer liver metastasis, Neoadjuvant chemotherapy resistance, Spatial immune barrier, *SPP1* + macrophages, CD8 + T cell exhaustion

## Abstract

Neoadjuvant chemotherapy (NAC) for colorectal cancer liver metastasis (CRLM) remains a major clinical challenge, with outcomes varying widely among patients, yet the mechanisms that drive therapeutic resistance remain incompletely understood. In this study, we integrated single‑cell RNA‑sequencing and spatial transcriptomics of patient tumor specimens to dissect the interplay between the hepatic tumor microenvironment and chemotherapy resistance. We identified a specific macrophage subpopulation marked by elevated expression of Secreted Phosphoprotein 1 (*SPP1*) that is significantly enriched in insufficient responders to NAC. Mechanistically, these *SPP1* + macrophages exhibit a pro‑tumoral transcriptomic profile and directly suppress CD8 + T cell cytotoxic function through the *SPP1‑CD44* signaling axis, as demonstrated by in *vitro* co‑culture assays, a large‑scale tissue microarray, and multiple syngeneic mouse models. Spatially, they are scattered throughout the tumour and form dense barriers that sequester CD8 + T cells and drive their exhaustion. Strikingly, this spatial configuration inversely correlates with cytotoxic activity and adaptive immune signatures and thereby reinforces a local immunosuppressive niche that sustains chemoresistance. These data provide the missing link: effective chemotherapy requires an immune‑active hepatic microenvironment, and *SPP1* + macrophage infiltration actively disrupts this state, which in turn promotes clinical resistance. In a preclinical CRLM model, SPP1 blockade synergised with anti‑PD‑1, inducing significant tumour regression and restoring T‑cell function. Collectively, our findings position *SPP1* + macrophages as central drivers of NAC resistance in CRLM and highlight the spatial immune barrier they create as a promising therapeutic target for overcoming chemoresistance and improving immunotherapy responses.

**Supplementary Information:**

The online version contains supplementary material available at https://doi.org/10.1186/s43556-026-00581-7.

## Introduction

Colorectal cancer (CRC) is a leading cause of cancer-related mortality worldwide, with the liver being the most common site of metastasis [1]. For patients with colorectal cancer liver metastasis (CRLM), neoadjuvant chemotherapy (NAC), often with oxaliplatin-based regimens, represents a critical strategy to downstage tumors and increase the likelihood of curative resection [2]. However, the clinical benefit of NAC is highly variable. This clinical challenge underscores a fundamental gap in our understanding: how does NAC, a cytotoxic intervention, paradoxically contribute to a more aggressive, therapy-resistant state in some patients?

The tumor microenvironment (TME) is increasingly recognized as a key determinant of therapeutic outcomes [3]. The TME is a complex ecosystem of cancer cells, stromal cells, and a diverse array of immune cells [3, 4].Among these, tumor-associated macrophages (TAMs), originating from both tissue-resident precursors (like liver Kupffer cells) and circulating monocytes, are the most abundant immune population and are known for their profound functional plasticity [5]. While some TAMs can exert anti-tumoral functions, they are often "educated" by the TME to adopt a pro-tumoral, M2-like phenotype, thereby promoting tumor growth, angiogenesis, and immunosuppression [6]. The impact of NAC on the composition and functional state of TAMs in CRLM, and how this relates to treatment failure, remains largely uncharacterized.

Recent advances in single-cell genomics have begun to unravel the heterogeneity of TAMs, revealing distinct subpopulations with specialized roles [7]. While *SPP1* + macrophages have emerged as a pro-tumoral population in several cancers [8]. Secreted Phosphoprotein 1 (SPP1), also known as osteopontin, is a multifunctional secreted glycoprotein involved in immune regulation and tissue remodeling. Their precise identity and function within the NAC-treated CRLM microenvironment remain a critical black box. This knowledge gap leaves three fundamental questions unanswered: First, are *SPP1* + macrophages the principal cellular drivers of NAC resistance, or merely bystanders? Second, by what molecular mechanisms do they orchestrate immunosuppression? And third, what are their developmental origins under the selective pressure of chemotherapy? Addressing these questions is paramount to shifting the paradigm from simply observing resistance to rationally designing therapies to overcome it.

Here, we hypothesized that an insufficient response to NAC is driven by the emergence of a specific, immunosuppressive macrophage subpopulation that actively contributes to therapeutic failure. We aimed to identify this subpopulation, elucidate the molecular mechanisms by which it suppresses anti-tumor immunity, and validate its functional role and therapeutic actionability. Through a comprehensive, multi-platform approach combining single-cell and spatial transcriptomics of patient tumor specimens with functional in *vitro* and in *vivo* models, we identified *SPP1* + macrophages as correlates of insufficient response to NAC in CRLM. Since chemotherapy efficacy depends on establishing an immune‑active TME via immunogenic cell clearance, an immunosuppressive stroma can subvert this effect. We bridge this gap by showing that *SPP1* + macrophage‑enriched stroma drives immune exclusion and CD8 + T cell dysfunction, thereby conferring chemoresistance.

## Results

### Neoadjuvant chemotherapy reshapes the immune microenvironment in CRLM

**Fig. 1:**
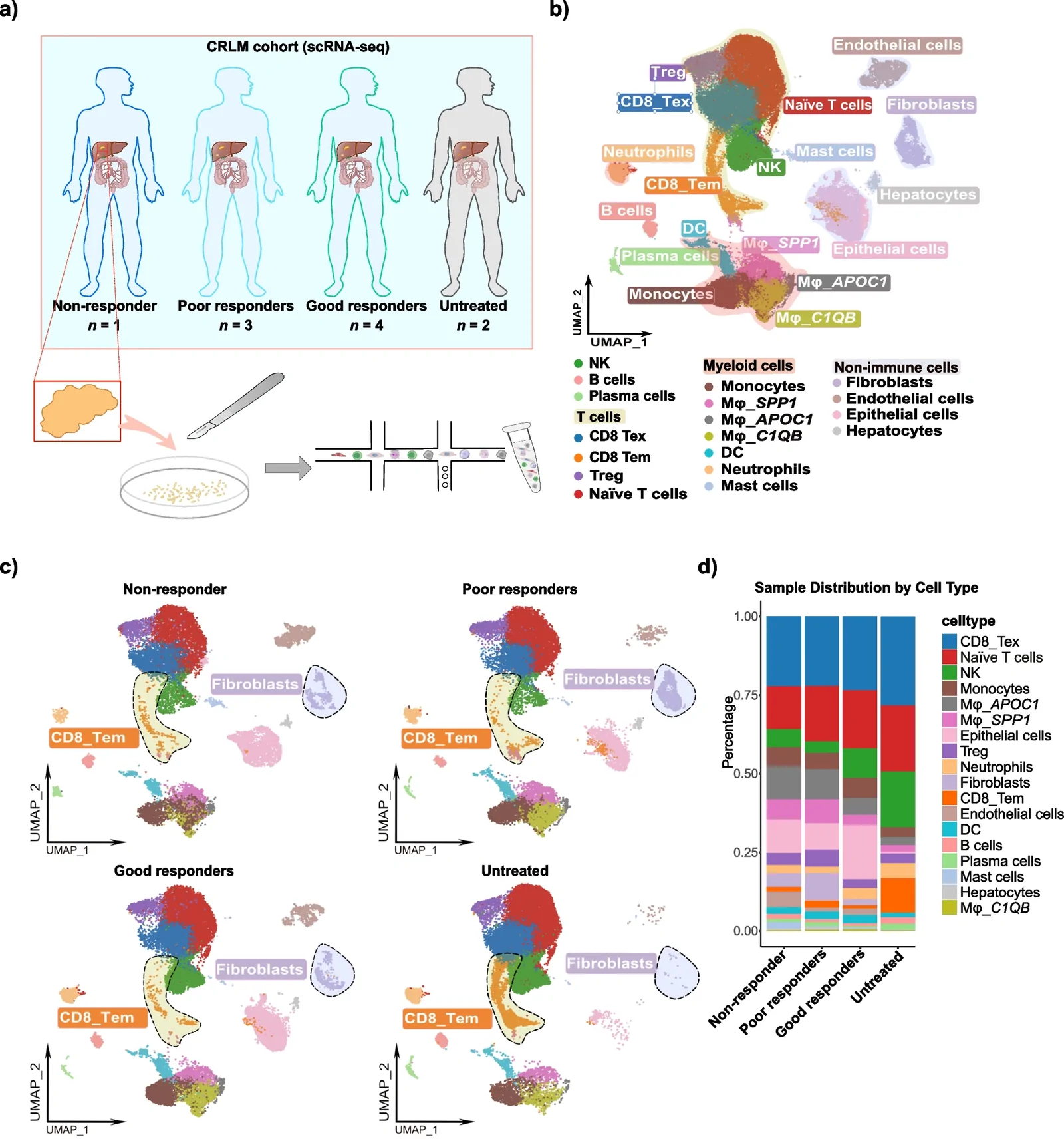
Neoadjuvant chemotherapy reshapes the immune microenvironment in CRLM. **a** Schematic workflow and sample composition of the primary single-cell RNA sequencing (scRNA-seq) cohort. Tumor specimens were obtained from a clinical cohort of 10 colorectal cancer liver metastasis (CRLM) patients stratified by therapeutic response, consisting of non-responder (*n* = 1), poor responders (*n* = 3), good responders (*n* = 4), and untreated treatment-naïve (*n* = 2) patients. **b** UMAP visualization of 99,694 single cells from 10 CRLM patients, colored by 18 annotated cell types. Cell type annotation was based on canonical marker genes shown in Fig. S1a. **c** UMAP plots showing the distribution of cell types across the four patient groups. Note the visual enrichment of macrophages (Mφ) and fibroblasts in non- and poor responders groups. **d** Stacked bar plot quantifying the proportional distribution of annotated cell types across the four patient groups, confirming the compositional changes observed in (**c**)

To determine whether the cellular composition of the tumour microenvironment differs between patients who respond or do not respond to NAC, we performed single‑cell RNA‑sequencing (scRNA‑seq) on surgical specimens from 10 CRLM patients. These were grouped into good responders, poor responders, non‑responders, and untreated patients based on pathological response criteria (Fig. 1a). After quality filtering, we obtained 99,694 cells and clustered them using an unbiased graph‑based approach. We identified 18 major cell types, covering myeloid, lymphoid, stromal, and epithelial compartments, and annotated each cluster with established marker genes (Fig. 1b; Fig. S1a). When we compared the relative proportions of these populations across groups, a consistent pattern emerged: the non‑responder and poor‑responder groups both showed a marked increase in macrophages and fibroblasts compared with good responders and untreated patients (Fig. 1c, d). No other cell type showed a similarly consistent shift. These observations suggest that an insufficient pathological response to NAC is associated with a relative expansion of myeloid and fibroblast populations, pointing to a more fibrotic and immunosuppressive microenvironment in tumours that escape chemotherapy.

### SPP1 + macrophages are a key feature distinguishing poor from good responders to NAC

**Fig. 2:**
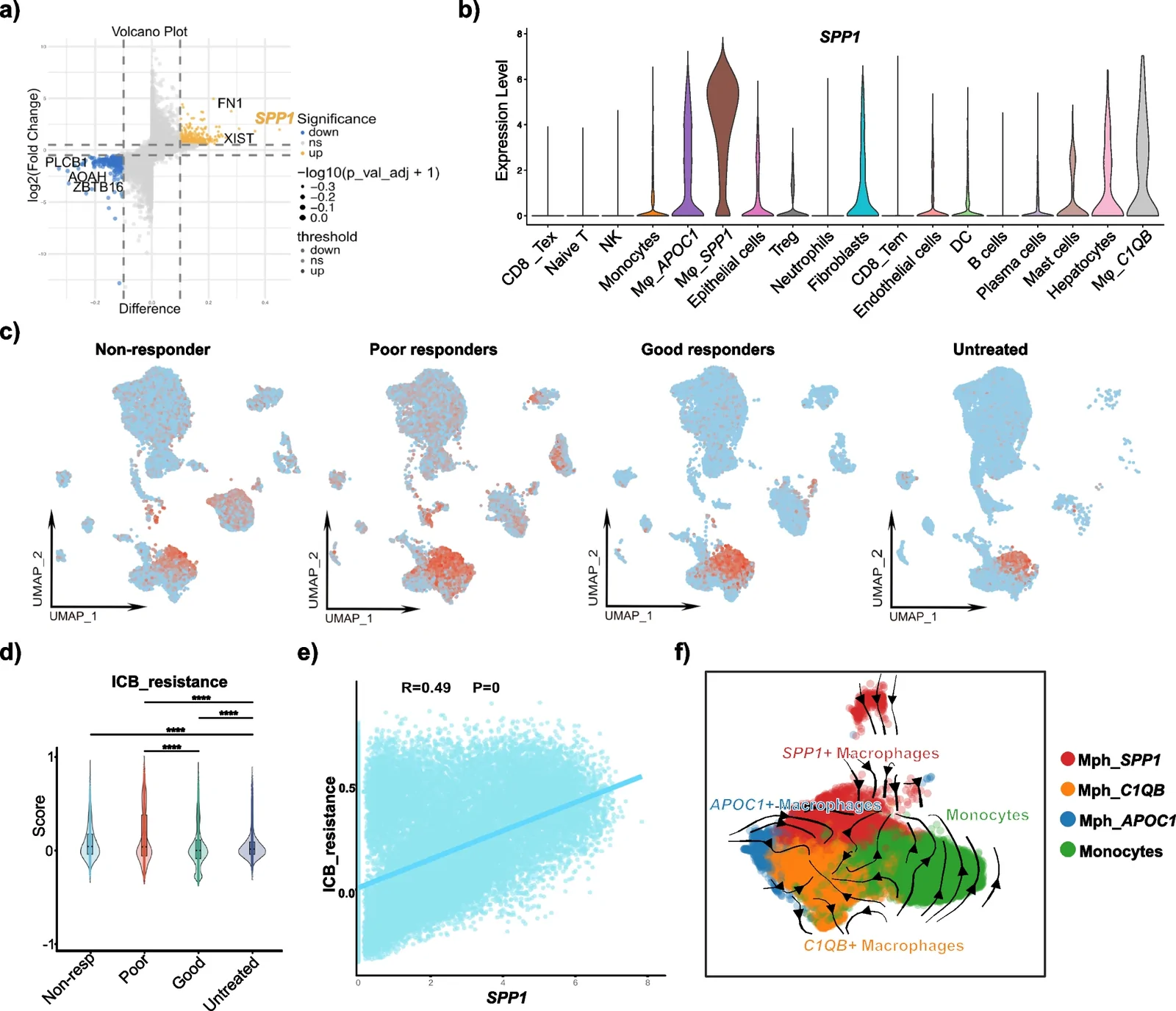
*SPP1* + macrophages are a key pro-tumoral subpopulation defining the "insufficient responder" TME. **a** Volcano plot showing differentially expressed genes in macrophages between poor responders and good responders. *SPP1* is highlighted as a top upregulated gene. **b** Violin plots illustrating the normalized expression of *SPP1* across all major cell types, demonstrating its predominant expression in macrophage subpopulations (Mφ_*SPP1*, *Mφ_APOC1*, *Mφ_C1QB*). **c** UMAP plot showing the specific expression of the *SPP1* gene projected onto all cells, highlighting the Mφ_*SPP1* cluster. **d** Violin plots of Gene Set Variation Analysis (GSVA) scores for an immune checkpoint blockade (ICB) resistance signature, compared across patient groups. **e** Scatter plot showing the positive correlation between *SPP1* gene expression and the ICB resistance score across all single cells. R and *P* values from Pearson correlation are shown. **f** RNA velocity stream plot projected on the macrophage sub-clusters, with arrows indicating a clear developmental trajectory from monocyte toward the terminally differentiated *SPP1* + macrophage state. Statistical significance in (**d**) was determined by one-way ANOVA. ****p < 0.0001

To identify the molecular differences that distinguish poor from good responders, we compared the transcriptomes of macrophages isolated from these two groups. Differential gene expression analysis revealed that *SPP1*, encoding the secreted glycoprotein osteopontin, was among the most strongly upregulated genes in the poor‑responder group (Fig. 2a). Within the macrophage compartment, *SPP1* expression was largely restricted to a single cluster, which we term *SPP1* + macrophages (Fig. 2b, c). To further corroborate this finding at the global transcriptional level, we analysed the differential gene expression between liver metastasis and paired adjacent tissues. This analysis confirmed that *SPP1* was also one of the most significantly upregulated genes in metastatic lesions compared with adjacent liver tissues (Fig. S1b), reinforcing the clinical relevance of *SPP1* upregulation in the CRLM setting.

Functionally, these *SPP1* + macrophages were associated with a pro-tumoral phenotype. Gene set variation analysis (GSVA) revealed that they were significantly enriched for pathways related to Epithelial-Mesenchymal Transition (EMT) and Angiogenesis, while showing lower scores for Tertiary Lymphoid Structures (TLS), which are associated with favorable immune responses (Fig. S1c-g). Consistent with this, cells expressing high levels of *SPP1* also exhibited significantly higher scores for an immune checkpoint blockade (ICB) resistance signature, and *SPP1* expression positively correlated with this resistance score across the dataset (Fig. 2d, e). To explore their dynamic origins, we applied RNA velocity analysis, which strongly suggested a developmental trajectory originating from monocyte population and terminally differentiating into the *SPP1* + macrophage state (Fig. 2f). ogether, these data indicate that *SPP1* + macrophages represent a functionally distinct population, associated with pro‑tumoral pathways and features of immune resistance, and that they may arise from monocytic precursors under the selective pressure of NAC.

### The SPP1-CD44 axis mediates the suppression of CD8 + T cell function

**Fig. 3:**
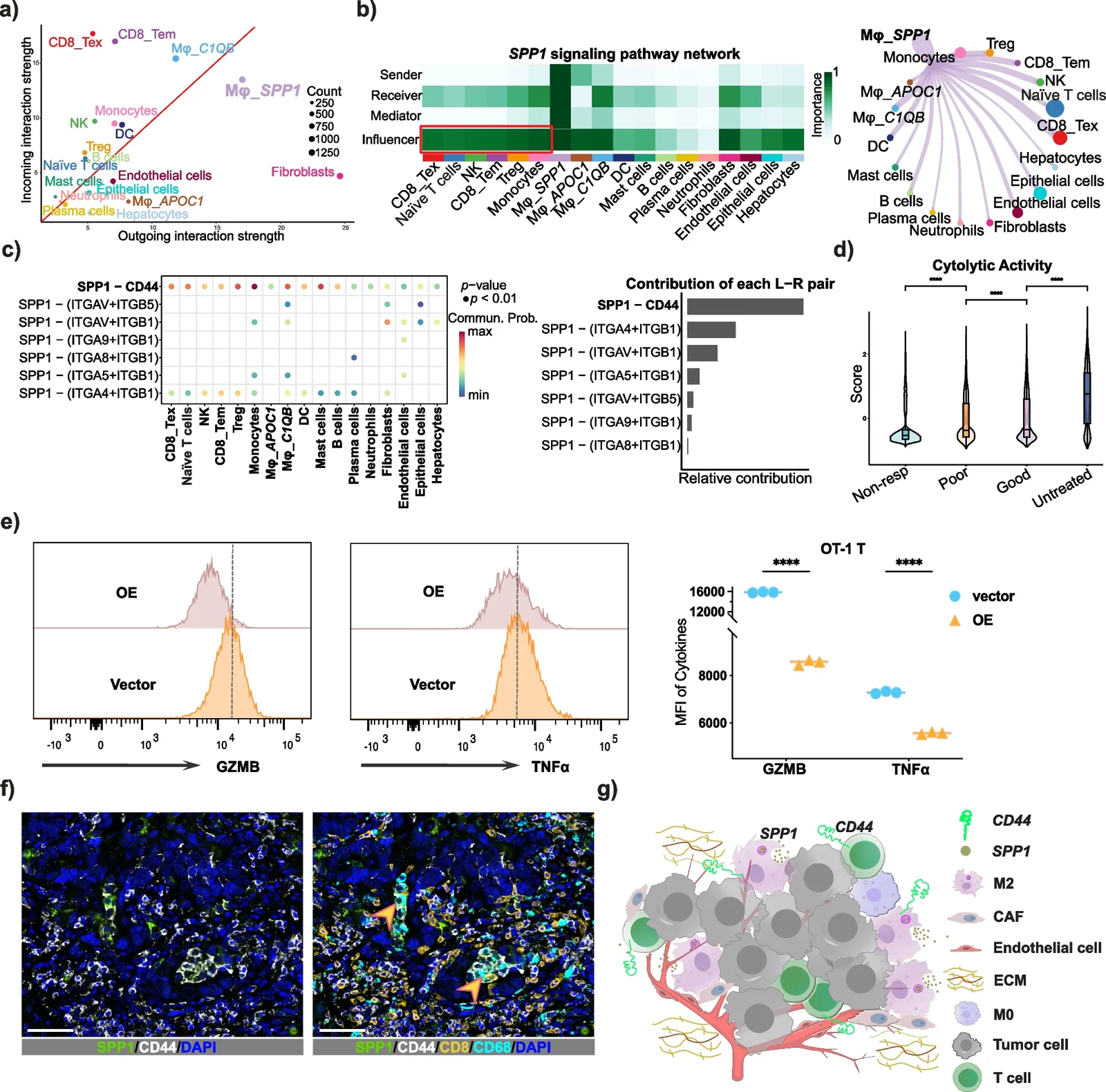
The SPP1-CD44 axis mediates the suppression of CD8 + T cell function by *SPP1* + macrophages. **a** Circle plot from CellChat analysis showing the overall strength and number of cell–cell interactions, highlighting Mφ_*SPP1* as a major signaling sender. **b** Heatmap illustrating the contribution of major signaling pathways originating from Mφ_*SPP1*, with the *SPP1* pathway being dominant (left). Visualization of signaling from Mφ_*SPP1* to other cell clusters (right). **c** Bubble plot showing the significance and contribution of major ligand-receptor pairs within the *SPP1* signaling pathway, identifying SPP1-CD44 as a primary interaction (left). SPP1-CD44 L-R pair makes the major contribution of *SPP1* signaling pathway (right). **d** Violin plots of GSVA scores for cytolytic activity across the four patient groups. **e** Representative flow cytometry plots and quantification of cytokine production (GZMB, TNFα) by activated OT-1 CD8 + T cells after co-culture with *SPP1*-overexpressing (OE) or vector control RAW264.7 macrophages. **f** Representative multiplex immunofluorescence (mIHC) image of a CRLM tissue section showing the spatial co-localization of SPP1 (green), CD44 (white), CD8 (yellow) and the macrophage marker CD68 (Cyan). Arrows indicate cells showing colocalization of the indicated markers. Scale bar: 50 μm. **g** Schematic illustration of the SPP1‑CD44 signaling axis within the CRLM immunosuppressive niche. SPP1 is predominantly secreted by M2‑like tumor‑associated macrophages and acts on CD44 receptors expressed on the surface of adjacent T cells, thereby contributing to local immune suppression and CD8 + T cell dysfunction. Statistical significance in (**d**) was determined by Kruskal–Wallis test, and in (**e**) by Student's t-test. ****p < 0.0001, ***p < 0.001

Given the pro-tumoral and immunosuppressive functional profile of *SPP1* + macrophages, we next investigated the molecular mechanisms by which they might impair anti-tumor immunity. To address this, we performed cell‑cell interaction analysis using CellChat on our scRNA‑seq data. This approach predicted that *SPP1* + macrophages act as dominant “sender” cells, with the *SPP1* signaling pathway representing a major outgoing communication route directed toward T cells (Fig. 3a, b). Within this network, the ligand‑receptor pair *SPP1‑CD44* emerged as a particularly strong interaction (Fig. 3c), and we confirmed this finding in an independent external dataset (Fig. S2a–c). We also observed that insufficient responders, which harbour more *SPP1* + macrophages, displayed lower cytotoxic activity scores in the TME (Fig. 3d; Fig. S2d), consistent with the idea that this pathway may suppress T‑cell function.

To test this directly, we set up an in *vitro* co‑culture system using OT-1 CD8 + T cells and *SPP1*-overexpressing RAW264.7 macrophages. Co‑culture with *SPP1*-overexpressing macrophages significantly reduced the production of effector cytokines, including GZMB and TNFα, by activated T cells (Fig. 3e). Finally, we examined patient CRLM tissues by multiplex immunofluorescence. This revealed that *SPP1* + macrophages (CD68 + SPP1 +) are physically close to CD44‑expressing cells within the tumour, supporting the spatial basis for the predicted ligand‑receptor interaction (Fig. 3f). Together, these findings indicate that *SPP1* + macrophages can engage the SPP1‑CD44 axis with neighbouring T cells (Fig. 3g), and that this interaction is associated with impaired T‑cell effector function.

### Spatial transcriptomics reveals the in situ architecture of the SPP1-driven immunosuppressive niche

**Fig. 4:**
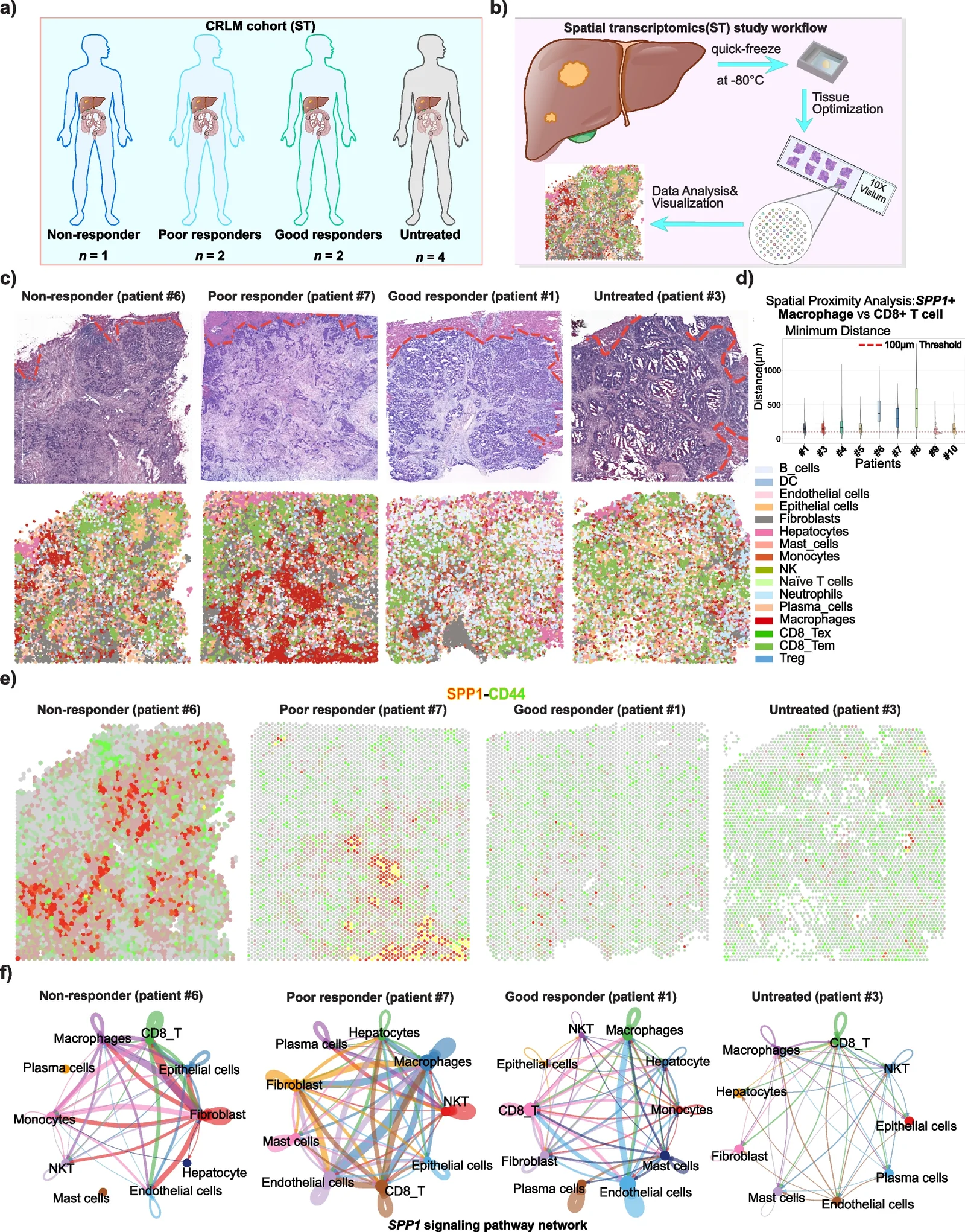
Spatial transcriptomics reveals the in situ architecture of the *SPP1*-driven immunosuppressive niche. **a** The composition of CRLM patient in spatial transcriptome sequencing, including 1 non-responder, 2 poor responders, 2 good responders and 4 untreated from patients with patients with liver metastases after colorectal cancer surgery. **b** Schematic of spatial transcriptomics (ST) study workflow. **c** Representative spatial transcriptomics (ST) data from non-responder, poor responders, good responders, and Untreated CRLM patients. For each patient, H&E staining (top) and corresponding cell-type deconvolution maps generated by CytoSPACE (bottom) are shown, revealing in situ enrichment of macrophages in insufficient responders. **d** Neighborhood enrichment analysis plot quantifying the spatial interaction between *SPP1* + macrophages and CD8 + T cells across all ST samples. A score < 1 indicates significant spatial exclusion. **e** Visualization of normalized SPP1 (red) and CD44 (green) gene expression on a representative ST slide from an insufficient responder, demonstrating their spatial co-localization. **f** Spatial neighborhood graph network for *SPP1* signaling pathway in representative patients from each group, showing stronger communication networks in non- and poor-responders

To examine the spatial organization of *SPP1* + macrophages and CD8 + T cells within the native tissue architecture, we performed spatial transcriptomics (ST) on CRLM sections (Fig. 4a, b). By integrating our scRNA‑seq data as a reference, we deconvolved the cell type composition of each spatial spot and confirmed that tumour regions from insufficient responders contained markedly more *SPP1* + macrophages, whereas CD8 + T cells appeared less abundant in these areas (Fig. 4c, Fig. S3a-c). To quantify this spatial relationship, we performed neighbourhood analysis, which revealed a significant spatial exclusion between *SPP1* + macrophages and CD8 + T cells—in other words, the two populations were consistently farther apart in insufficient responders than in good responders (Fig. 4d). In the same tissue sections, *SPP1* + macrophages often formed dense clusters that appeared to physically separate CD8 + T cells from tumour cells, suggesting a structural basis for T‑cell exclusion. This pattern was observed consistently across all insufficient responders, including patients 6, 7, and 8. In addition, we examined the spatial distribution of *SPP1* and *CD44* expression. These two signals showed strong co‑localization within the same regions, indicating that the ligand and its receptor are present in close proximity in situ (Fig. 4e). We then asked whether the *SPP1* signaling network is more active in insufficient responders. Spatial communication analysis confirmed that *SPP1*‑related interactions were significantly stronger in non‑responders and poor responders than in good responders (Fig. 4f). In the same groups, we also observed enrichment of angiogenesis markers such as *PECAM1* (Fig. S4a), while macrophages in these tumours displayed reduced expression of immune‑related gene sets, including interferon_α_response, interferon_γ_response, and inflammatory response pathways (Fig. S4b). Together, these spatial data demonstrate that *SPP1* + macrophages are not only transcriptionally distinct but also organised into physical barriers that may contribute to CD8 + T cell exclusion and to a locally suppressed immune environment in NAC‑refractory lesions.

### High SPP1 expression is a robust prognostic biomarker for poor outcomes in CRC

**Fig. 5:**
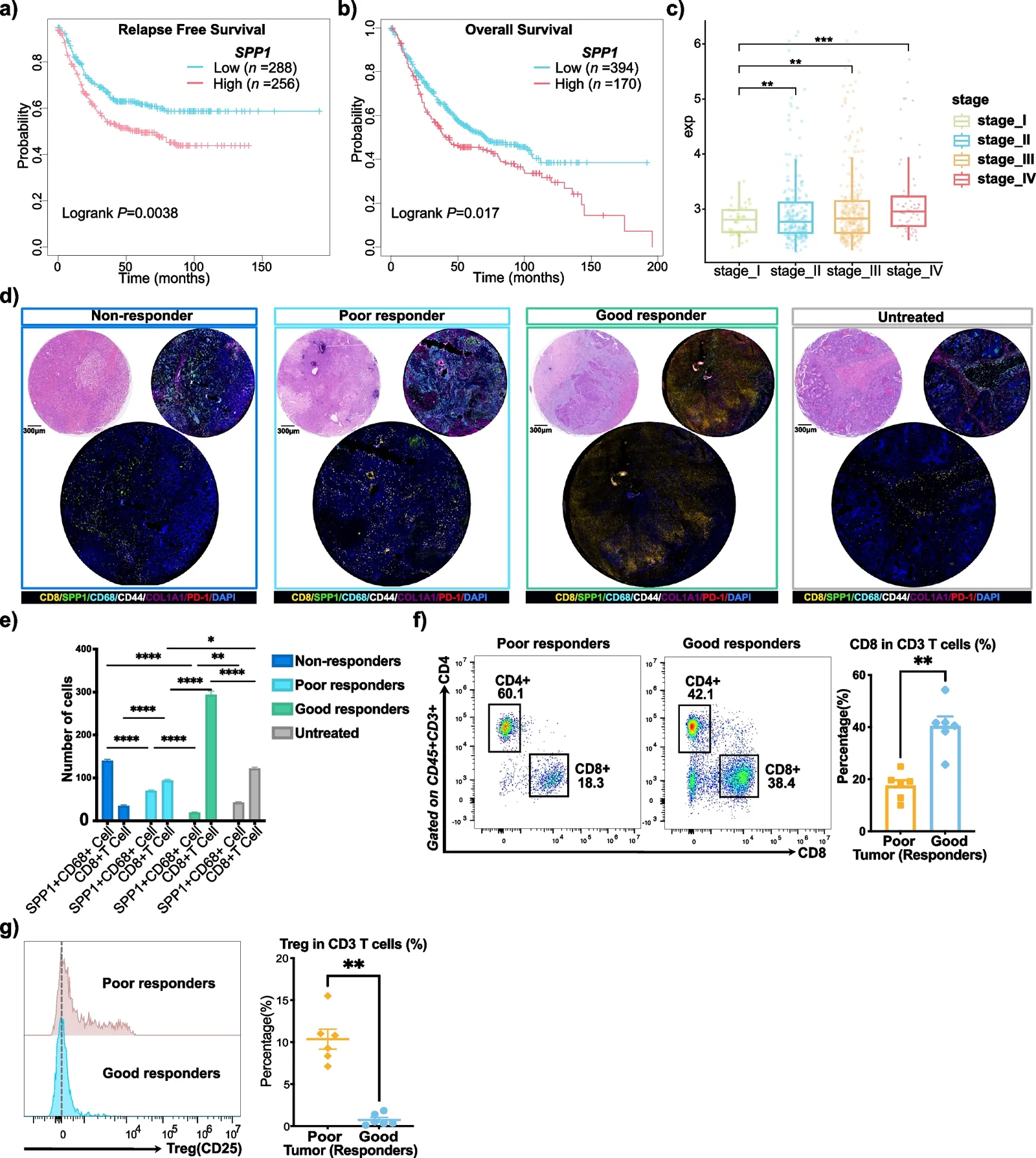
High *SPP1* expression is a robust prognostic biomarker for poor outcomes in CRC. **a**, **b** Kaplan–Meier curves for relapse-free survival (RFS) (**a**) and overall survival (OS) (**b**) in the colorectal cancer cohort from the GEO meta-dataset (analyzed via Kaplan–Meier Plotter), stratified by high versus low *SPP1* gene expression. *P*-values were calculated using the log-rank test. **c** *SPP1* expression in colorectal cancer tissues across different pathological stages. Data were obtained from the Gene Expression Omnibus (GEO) database. **d** Representative mIHC images from the CRLM tissue microarray (TMA, *n* = 30) showing staining for CD8 (yellow), SPP1 (green), CD68 (cyan), and PD-1 (red) in patients from different response groups. Scale bar: 300 μm. **e** Quantification of *SPP1* + *CD68* + macrophage density and CD8 + T cell density from the TMA analysis, compared across patient response groups. **f** Flow cytometry (FACS) analysis revealed significant differences in CD8 + T cell populations between poor responders and good responders (*n* = 12). All the samples were derived from freshly resected human CRLM tumor tissues. **g** FACS analysis showing that the difference in Treg between poor responders and good responders (*n* = 12). All the samples were derived from freshly resected human CRLM tumor tissues. Statistical significance in (**e**) was determined by one-way ANOVA. **p < 0.01, ***p < 0.001, ****p < 0.0001

To evaluate whether our findings extend to larger patient populations, we first examined public bulk RNA‑seq datasets. In a GEO colorectal cancer cohort, high *SPP1* expression was associated with shorter relapse‑free and overall survival (Fig. 5a, b), and *SPP1* levels increased with advancing pathological stage (Fig. 5c). We then asked whether the same relationship holds at the protein level in our own patient material. To address this, we constructed a tissue microarray (TMA) from 30 CRLM patients and performed multiplex immunofluorescence staining. Automated image analysis showed that insufficient responders had a significantly higher density of *SPP1* + *CD68* + macrophages than good responders, while CD8 + T cell infiltration was correspondingly reduced in the same patients (Fig. 5d, e). To further corroborate these findings, we analysed an independent cohort of freshly resected CRLM specimens by flow cytometry. This confirmed that poor responders had a lower CD8 + T cell frequency and a higher Treg proportion compared with good responders (Fig. 5f, g). Together, these three independent lines of evidence—public transcriptomic data, our TMA cohort, and an external flow cohort—consistently link *SPP1* + macrophage abundance with poor prognosis, advanced disease, and an immunosuppressive T‑cell landscape in CRLM.

### Macrophage-derived SPP1 is sufficient to drive tumor progression and immune suppression in vivo

**Fig. 6:**
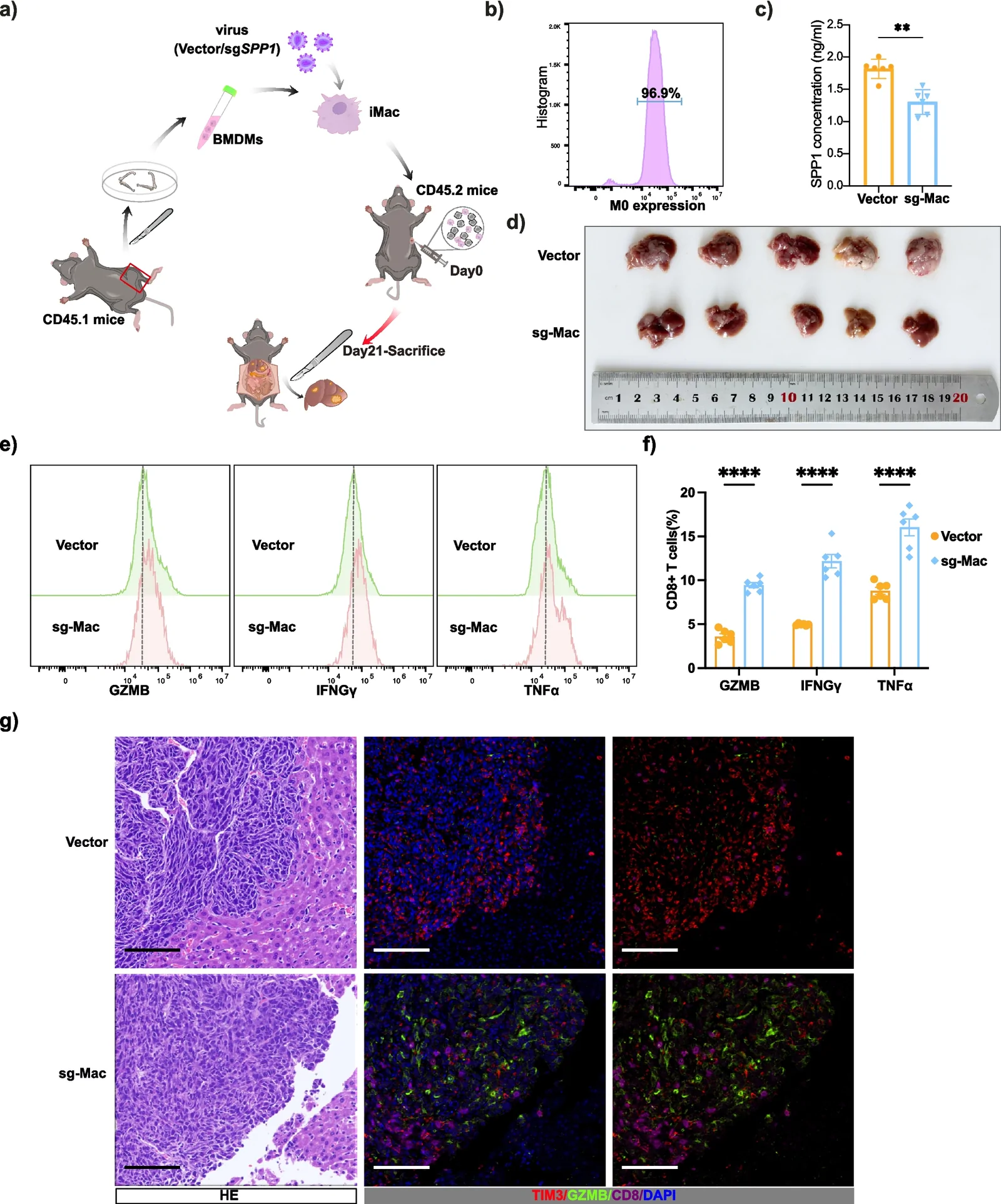
Macrophage-derived *SPP1* is sufficient to drive tumor progression and immune suppression in *vivo*. **a** Schematic of the adoptive transfer experiment. Bone marrow-derived macrophages (BMDMs) from CD45.1 mice were transiently transfected with a CRISPRi plasmid encoding dCas9 and an SPP1‑targeting sgRNA (sg‑Mac) or a control plasmid, then co-injected with MC38 tumor cells into CD45.2 recipient mice. **b** Flow cytometry histogram confirming successful transduction and M0 expression in BMDMs. **c** ELISA quantification showing reduced SPP1 protein secretion from sg-Mac cells. **d** Representative images of livers and tumors harvested on day 21 post-implantation. **e**, **f** Flow cytometry quantification of cytokine-producing (GZMB, IFNγ, TNFα) CD8 + T cells from the tumors of recipient mice. **g** Representative H&E and immunofluorescence images of tumor sections. Scale bar: 100 μm. Statistical significance was determined by Student's t-test or one-way ANOVA. **p < 0.01, ****p < 0.0001

To determine whether *SPP1* derived specifically from macrophages directly promotes tumour growth and immune suppression in CRLM, we performed an adoptive transfer experiment using a loss‑of‑function approach. Bone marrow‑derived macrophages (BMDMs) from CD45.1 donor mice were first isolated and differentiated, and their purity and phenotype were confirmed by flow cytometry (Fig. 6a, b). The cells were then transiently transfected with either an *SPP1*‑knockdown plasmid (sg‑Mac) or a control plasmid. To verify knockdown efficiency, we measured SPP1 secretion in the culture supernatant by ELISA, which confirmed a significant reduction in extracellular SPP1 levels in the sg‑Mac group compared with vector (Fig. 6c). These macrophages were then co‑injected with MC38 tumour cells into CD45.2 recipient mice. Three weeks later, mice that received *SPP1*‑knockdown macrophages showed substantially lower tumour burden than those given vector macrophages (Fig. 6d). To understand the immune basis of this difference, we analysed tumour‑infiltrating lymphocytes by flow cytometry. The sg‑Mac group showed a higher frequency of CD8 + T cells producing effector cytokines, including GZMB, IFNγ, and TNFα, compared with the vector group (Fig. 6e, f). These flow cytometry findings were further supported by multiplex immunohistochemistry (mIHC) on tumour sections. The sg‑Mac group exhibited increased GZMB and CD8 expression, whereas the vector group showed higher Tim3 levels, indicating that *SPP1* knockdown reduces T‑cell exhaustion and promotes cytotoxic activity within the TME (Fig. 6g). Together, these data provide direct in *vivo* evidence that macrophage‑derived *SPP1* contributes to tumour progression and local immune suppression, and that its knockdown is sufficient to restore CD8 + T cell effector function within the TME.

### Dual blockade of SPP1 and PD-1 synergistically inhibits tumor growth and reinvigorates anti-tumor immunity

**Fig. 7:**
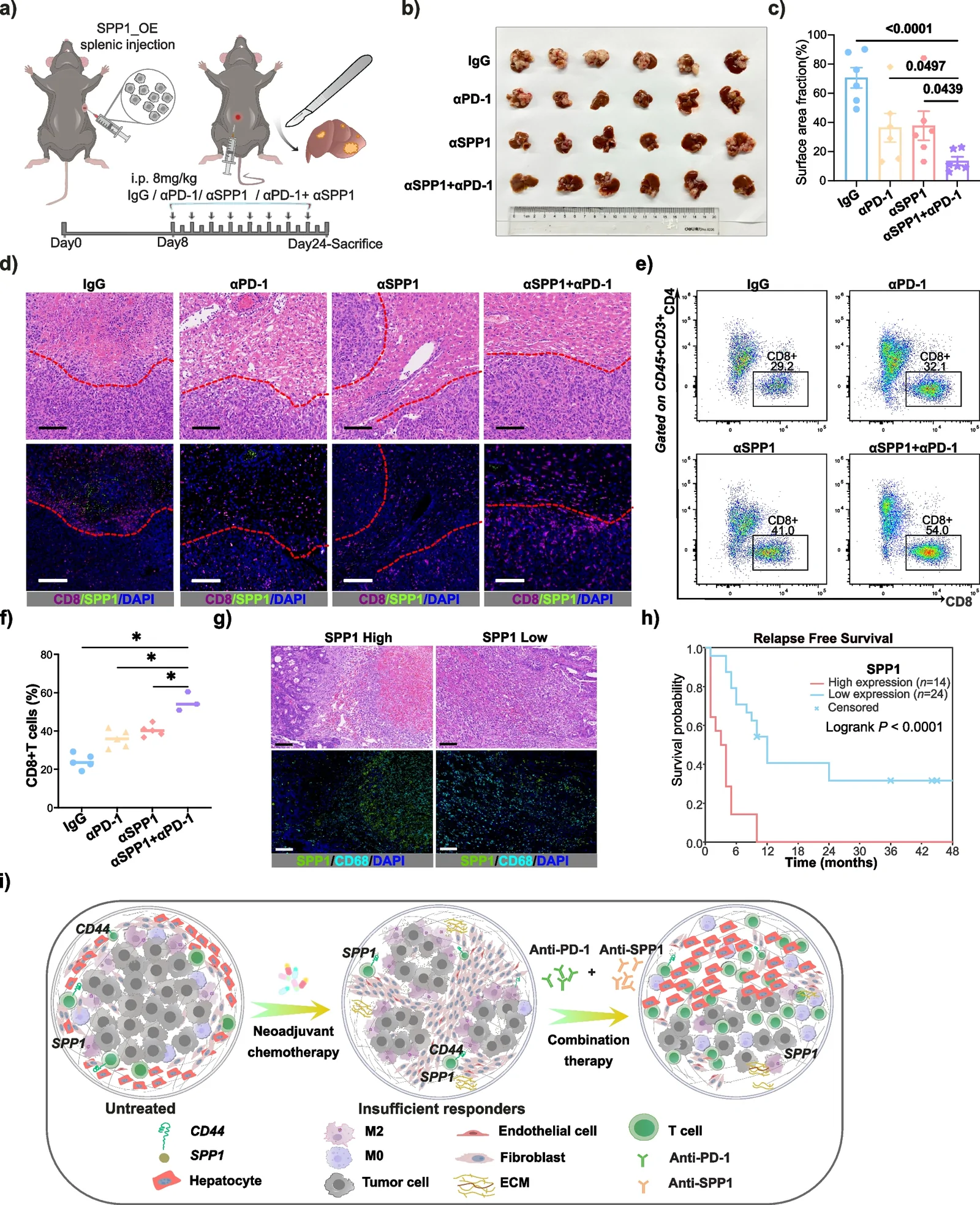
Dual blockade of SPP1 and PD-1 synergistically inhibits tumor growth and reinvigorates anti-tumor immunity. **a** Schematic of the combination therapy experiment in the MC38-*SPP1*-OE CRLM model. **b** Representative images of livers and tumors harvested on day 24 after treatment initiation. **c** Quantitative analysis of the macroscopic tumor surface area fraction (expressed as tumor area / total liver area, %) across the designated treatment groups at Day 24, analyzed via ImageJ. Data represent mean ± SEM (*n* = 6). Statistical significance was determined by one-way ANOVA. **** *P* < 0.0001. **d** Representative H&E and mIHC images showing CD8 + T cell (purple) and SPP1 (green) expression in tumors from the four treatment groups. Dotted lines delineate the tumor boundary. Scale bar: 100 μm. **e** Representative flow cytometry plots of tumor-infiltrating CD45 + CD3 + T cells, gated for CD8 + populations. **f** Flow cytometry analysis of tumor-infiltrating immune cells across the four designated treatment groups. For the combination treatment group, due to profound tumor regression and insufficient cell yield from single animals, harvested tumor tissues from every two individual mice were pooled together prior to cell dissociation to meet the baseline cellular requirements for flow cytometry. Thus, the data points in the Combo group represent *n* = 3 independent pooled analytical samples derived from a total of *n* = 6 treated mice. Data represent mean ± SEM. **g** Representative immunohistochemical images of SPP1 staining in resected liver metastasis specimens from patients with high and low SPP1 expression. Scale bar: 100 μm. SPP1 (green), CD68 (cyan), and DAPI (blue). **h** Kaplan–Meier survival analysis of recurrence‑free survival (RFS) stratified by SPP1 expression level (median IHC score as cutoff). The log‑rank test was used for comparison (*p* < 0.0001). All patients in this cohort (*n* = 38) received uniform post‑operative chemotherapy after surgical resection. **i** Schematic summary of the SPP1‑mediated immunosuppressive mechanism and combination therapy in CRLM. Statistical significance was determined by one-way ANOVA. *p < 0.05, **p < 0.01, ****p < 0.0001

Given that *SPP1* + macrophages contribute to an immunosuppressive barrier, we next asked whether therapeutic targeting of *SPP1* could sensitize tumours to immune checkpoint blockade. To address this in a clinically relevant setting, we used an MC38‑based CRLM model with forced *SPP1* overexpression. Naive wild‑type MC38 tumours express negligible baseline *SPP1* and do not reproduce the immunosuppressive microenvironment typical of chemoresistant human disease; the *SPP1*‑OE model therefore serves as a stringent preclinical surrogate that mimics the high‑*SPP1* niche observed in insufficient responders. Mice bearing established *SPP1*‑OE tumours were randomized to receive isotype control, anti‑SPP1 alone, anti‑PD‑1 alone, or the combination (Fig. 7a). Tumour growth was monitored and, at endpoint, liver sections were photographed for macroscopic tumour area quantification using ImageJ. Anti‑SPP1 or anti‑PD‑1 alone produced only modest tumour control, whereas the combination led to a marked and synergistic reduction in tumour burden (Fig. 7b). Digital quantification of the tumour surface area fraction confirmed this effect: the combination reduced the tumour‑to‑liver area ratio to approximately 13%, compared with roughly 70% in the isotype control group (Fig. 7c). To examine whether this therapeutic benefit was accompanied by immune remodelling, we performed multiplex immunohistochemistry and flow cytometry on tumour tissues. Both approaches revealed that only the combination group showed a substantial increase in CD8 + T cell infiltration (Fig. 7d-f). These data indicate that anti‑SPP1 and anti‑PD‑1 act synergistically to reduce tumour growth in a high‑SPP1 microenvironment, and that this effect is associated with enhanced CD8 + T cell recruitment. Together, these findings support the rationale for combining SPP1 blockade with PD‑1 inhibition as a potential strategy for CRLM refractory to conventional therapy.

### High SPP1 expression predicts early relapse in CRLM patients receiving post-operative chemotherapy

To evaluate the clinical relevance of SPP1 in chemotherapy resistance and postoperative disease progression, we analyzed a retrospective cohort of 38 CRLM patients who underwent surgical resection followed by uniform adjuvant chemotherapy. Immunohistochemical (IHC) staining of resected liver metastasis specimens revealed notable inter‑patient heterogeneity in SPP1 expression levels, with staining intensities ranging from negative to strong (Fig. 7g). Using the median IHC score as the cutoff, patients were stratified into SPP1‑high and SPP1‑low groups. Kaplan–Meier survival analysis showed that SPP1‑high patients had significantly shorter relapse‑free survival (RFS) compared with SPP1‑low patients (log‑rank *p* < 0.0001; HR = 5.16, 95% CI 2.30–11.82; Fig. 7h). Since all patients in this validation cohort received post‑operative adjuvant chemotherapy, these clinical data suggest that elevated SPP1 expression is associated with a poor chemotherapy response and earlier relapse after adjuvant treatment in human CRLM.

Based on the integrated findings from our single‑cell and spatial transcriptomic analyses, together with functional in *vitro* and in *vivo* experiments, we proposed a working model for the SPP1‑driven immunosuppressive niche and its therapeutic targeting in CRLM (Fig. 7i). In patients with insufficient response to neoadjuvant chemotherapy, *SPP1* + macrophages accumulate within the hepatic tumor microenvironment and, through the SPP1‑CD44 axis, promote CD8 + T cell exclusion and exhaustion, sustaining an immune‑resistant state. Dual blockade of SPP1 and PD‑1 synergistically dismantles this immunosuppressive barrier, restores CD8 + T cell effector function, and remodels the tumor microenvironment toward an immune‑active state, thereby overcoming chemoresistance and improving immunotherapy efficacy.

## Discussion

Neoadjuvant chemotherapy remains a cornerstone of treatment for colorectal cancer liver metastases, yet a considerable number of patients derive limited benefit and experience early relapse [9, 10]. The mechanisms that distinguish effective from ineffective responses have remained poorly defined, particularly regarding the role of the local immune microenvironment. Our study addresses this gap by identifying *SPP1* + macrophages as a central cellular component of the therapy‑resistant TME in patients who respond poorly to NAC [11]. Using a combination of single‑cell and spatial transcriptomics, we show that these cells are enriched in insufficient responders, carry a pro‑tumoral transcriptional programme, and physically segregate from CD8 + T cells within the tumour. Functionally, they suppress T cell cytotoxicity through the SPP1‑CD44 axis, and therapeutic blockade of this pathway restores immune activity and synergises with PD‑1 inhibition in preclinical models [12–15].

A distinctive feature of our study design is the emphasis on comparing poor responders with good responders, rather than a simple binary distinction between responders and non‑responders. This approach allowed us to interrogate the biology of suboptimal response more precisely, isolating mechanisms that operate during treatment adaptation rather than those that reflect intrinsic, pre‑existing resistance. Within this framework, *SPP1* + macrophages emerged as the primary cellular differentiator. Their enrichment was not merely a correlate of established resistance but was consistently observed across all insufficient responders, suggesting that they actively contribute to the transition towards therapeutic failure [16, 17]. This conclusion is reinforced by the spatial data: in tumour sections from poor responders, *SPP1* + macrophages formed dense clusters that correlated with CD8 + T cell exclusion and with reduced cytotoxic and inflammatory gene signatures, indicating that the spatial organisation of these cells reinforces a locally immunosuppressive niche [18–20].

Our mechanistic work identifies the SPP1‑CD44 axis as the principal pathway through which *SPP1* + macrophages impair T‑cell function. This interaction, predicted by CellChat and validated in *vitro* and in *vivo*, appears to act as a key checkpoint in the TME. We found that *SPP1* overexpression in macrophages reduced the production of effector cytokines by CD8 + T cells, and that adoptive transfer of *SPP1*‑knockdown macrophages significantly reduced tumour growth and restored T‑cell effector function. These findings are consistent with earlier reports implicating *SPP1* in tumour progression, but our study provides a more precise cellular and spatial context: the ligand is produced predominantly by a distinct macrophage subset, and its receptor is engaged in spatially confined niches that exclude T cells [21–24]. The combination of spatial transcriptomics and functional data further strengthens this interpretation, as the SPP1‑CD44 interaction was most active in regions where macrophages and T cells were physically separated, and where *SPP1* expression inversely correlated with cytotoxicity scores. While we focused on the CD44 receptor, we cannot exclude contributions from other SPP1‑binding partners such as integrins, which may also play a role in this context.

The translational implications of our findings are twofold. First, the density of *SPP1* + macrophages may serve as a predictive biomarker. Quantifying this population in pretreatment biopsies could help identify patients unlikely to benefit from standard NAC, allowing earlier consideration of alternative or combination regimens [25–28]. Second, our preclinical data provide a clear rationale for combining SPP1 blockade with PD‑1 inhibition. In the SPP1‑overexpressing CRLM model, dual therapy produced a synergistic reduction in tumour burden and a marked increase in CD8 + T cell infiltration. This effect is consistent with a model in which anti‑SPP1 dismantles the macrophage‑driven barrier, converting an immunologically excluded tumour into one permissive for checkpoint blockade. The spatial evidence that *SPP1* + macrophages form physical barriers separating T cells from tumour cells further supports this interpretation and highlights the importance of targeting both the molecular and structural components of immune suppression.

A major challenge in CRLM management is post‑operative disease recurrence, which is frequently driven by resistance to adjuvant chemotherapy. In this study, our single‑cell and spatial transcriptomic analyses revealed that *SPP1* + macrophages are enriched within the immunosuppressive tumor microenvironment of NAC‑insufficient patients. To translate these mechanistic findings into clinical relevance, we validated SPP1 expression in an independent cohort of CRLM patients who received uniform post‑operative chemotherapy. Patients with high SPP1 expression experienced significantly earlier recurrence and shorter recurrence‑free survival compared with those with low expression. Collectively, these clinical data support the notion that SPP1 serves not only as a functional mediator of chemotherapy resistance, but also as a candidate biomarker for predicting relapse risk in CRLM patients following adjuvant treatment.

Several limitations should be considered. Our clinical transcriptomic data are cross‑sectional and cannot capture the dynamic evolution of the TME within individual patients over time. Although we validated key findings in independent cohorts and used functional models to establish causality, longitudinal studies with paired pre‑ and post‑treatment samples would be needed to define the temporal sequence of macrophage reprogramming during NAC. In addition, our in *vivo* experiments relied on an experimental metastasis model that bypasses primary tumour development. While this model allowed controlled evaluation of the hepatic TME, it does not fully recapitulate the complexity of spontaneous metastasis. Future studies using orthotopic models or patient‑derived organoid‑immune co‑culture systems would help address this gap. Finally, while our data firmly establish the functional importance of the SPP1‑CD44 axis, the downstream signalling events within T cells, such as those involving AKT, NF‑κB, or other pathways, remain to be characterised biochemically [29, 30]. Addressing these questions will be important for understanding the full mechanism of *SPP1*‑mediated immune suppression and for refining therapeutic strategies targeting this pathway.

## Conclusion

Together, these findings establish *SPP1* + macrophages as a critical driver of NAC resistance in CRLM. The comparative analysis between poor and good responders enabled us to dissect the adaptive evolution of resistance rather than simply capturing pre‑existing non‑response. Within this framework, *SPP1* + macrophages emerged as active architects of an immunosuppressive, spatially segregated TME, operating through the SPP1‑CD44 axis to exclude and exhaust CD8 + T cells. Integrating functional and spatial evidence positions these cells as a mechanistic bridge between chemoresistance and immune evasion. Therapeutic SPP1 blockade synergised with PD‑1 inhibition to remodel the TME and restore antitumour immunity. These preclinical findings were further supported by clinical validation in an independent post‑chemotherapy cohort, where high SPP1 expression correlated with significantly shorter recurrence‑free survival. Our study thus provides a mechanistic basis for patient stratification and supports the clinical evaluation of SPP1‑directed combination strategies in refractory CRLM.

## Materials and methods

### Patient Cohort

Patient response to neoadjuvant chemotherapy (NAC) was evaluated based on imaging assessments according to the Response Evaluation Criteria in Solid Tumors (RECIST; version 1.1) [31]. To specifically investigate the molecular drivers underlying the heterogeneity within partial responders, we focused our primary differential analyses on two distinct subgroups: "Good responders": Defined as patients exhibiting > 50% tumor regression after NAC (excluding complete response). "Poor responders": Defined as patients exhibiting 30%−50% tumor regression after NAC. For broader compositional analyses and to contextualize our findings within the full spectrum of clinical outcomes, patients with stable disease (SD) or progressive disease (PD) were classified as "Non-responders". In analyses where a clear distinction between favorable and unfavorable outcomes was required, the "Poor responders" and "Non-responders" were collectively grouped as "Insufficient responders". This grouping is supported by our clinical data, which demonstrates that patients in the "Insufficient responder" category share a similarly poor prognosis, characterized by a significantly higher early recurrence rate compared to "Good responders" (Table 1). The sample size for the retrospective IHC validation cohort (*n* = 38) was determined based on the expected difference in SPP1 expression levels between recurrence and non‑recurrence groups, with reference to previous studies reporting SPP1 as a significantly upregulated gene in colorectal cancer liver metastases. Considering a potential dropout rate of approximately 10‑15%, the final sample size was set at 38 patients. The remaining patients were excluded due to insufficient tissue quality or incomplete clinical follow‑up data. All statistical analyses were performed using R v4.5 and GraphPad Prism v9. Survival curves were estimated using the Kaplan‑Meier method and compared using the log‑rank test. Hazard ratios (HR) with 95% confidence intervals (CI) were calculated using univariate Cox proportional hazards regression. All specimens used in this study were obtained from Fudan University Shanghai Cancer Center (FUSCC).

**Table 1:** Clinical information on patients

	**untreated (*n* = 147)**	**non-responders (*n* = 33)**	**poor responders (*n* = 35)**	**good responders (*n* = 28)**	***p*-value**
**Age**					
< 50	24 (16.3%)	6 (18.2%)	7 (20.0%)	8 (28.6%)	0.494
≥ 50	123 (83.7%)	27 (81.8%)	28 (80.0%)	20 (71.4%)	
**Gender**					
female	49 (33.3%)	9 (27.3%)	15 (42.9%)	17 (60.7%)	0.0238
male	98 (66.7%)	24 (72.7%)	20 (57.1%)	11 (39.3%)	
**5-year survival status**					
Live	66 (44.9%)	5 (15.2%)	14 (40.0%)	12 (42.9%)	0.0181
Death	81 (55.1%)	28 (84.8%)	21 (60.0%)	16 (57.1%)	
**LFFS**					
Mean (SD)	20.7 (15.4)	12.4 (12.5)	13.4 (11.5)	20.6 (11.4)	0.00231
Median [Min, Max]	16.0 [1.00, 83.0]	7.00 [1.00, 59.0]	11.0 [1.00, 58.0]	20.0 [4.00, 42.0]	
**Recurrence (≤ 5 months)**					
Yes	15 (10.2%)	13 (39.4%)	6 (17.1%)	2 (7.1%)	< 0.001
No	132 (89.8%)	20 (60.6%)	29 (82.9%)	26 (92.9%)	
**Recurrence (≤ 12 months)**					
Yes	48 (32.7%)	23 (69.7%)	22 (62.9%)	10 (35.7%)	< 0.001
No	99 (67.3%)	10 (30.3%)	13 (37.1%)	18 (64.3%)	

### Single-cell RNA sequencing and data processing

Fresh tumor tissue specimens were surgically resected from a clinical cohort of 10 CRLM patients. Upon excision, tissues were immediately immersed in ice-cold RPMI 1640 medium (Gibco) and transported to the laboratory within 30 min to minimize cold ischemia artifacts. To prepare single-cell suspensions, specimens were thoroughly washed with sterile PBS, minced into approximately 1 mm^3^ fragments, and enzymatically dissociated using a Human Tumor Dissociation Kit (Miltenyi Biotec, Cat# 130–095–929) on a gentle MACS Dissociator according to the manufacturer's instructions. The mechanical and enzymatic program was executed at 37 °C for 45 min. The resulting homogenate was passed through a 70 μ m cell strainer (BD Biosciences) and centrifuged at 300 × g for 5 min at 4 °C. Contaminating erythrocytes were eliminated using red blood cell lysis buffer (Sigma-Aldrich), and cell debris or dead cells were subsequently depleted via a Dead Cell Removal Kit (Miltenyi Biotec). Finally, cell viability (> 85%) and concentration were evaluated using a Countess II Automated Cell Counter.

Single-cell RNA sequencing (scRNA-seq) was conducted via the 10 × Genomics Chromium Single Cell 3' Library & Gel Bead Kit following the manufacturer's protocols for library preparation and sequencing on the Illumina NovaSeq 6000 Sequencing System. The resulting sequencing reads were processed using the 10 × Genomics data pipeline, and the cell‒gene count matrix data were filtered and normalized via the Seurat package [32]. Harvesting and processing each sample independently led to a patient-level batch effect, with cells clustering predominantly by patient rather than by cell type. To address this, low-quality cells were filtered, and each sample was individually scaled using SCTransform. The Harmony algorithm was then applied to merge the samples, aiming to alleviate batch effects and improve the accuracy of cellular type clustering. The combined matrix underwent clustering using the Louvain algorithm, and two-dimensional visualization was achieved through PCA and UMAP. Differential gene expression analysis between clusters was performed using the MAST hurdle model in Seurat, with specific criteria for log fold change and minimum fractional expression thresholds [33].

To minimize the potential confounding effects of heterogeneous treatments on the observed immune differences, the three distinct neoadjuvant chemotherapy NAC regimens administered to the patients in the single-cell cohort were closely balanced between the "good responder" (*n* = 4) and "poor responder" (*n* = 3) groups. Detailed distribution of the specific regimens is provided in Table 1.

### Spatial transcriptomics analysis

ST experiments were conducted using the 10X Visium Spatial Gene Expression Kit following the manufacturer's tissue optimization and library preparation protocols [34]. Fresh tissue, immediately postresection, was embedded in Tissue-Tek O.C.T. compound and snap-frozen. RNA integrity was assessed using the PicoPure RNA Isolation Kit, and only samples with RNA integrity values exceeding 7 were utilized. Spatially barcoded glass slides with poly-T reverse transcription primers were used to mount 10 μm thick tissue sections. Brightfield imaging and H&E staining were performed, and after imaging, permeabilization was carried out to release and capture mRNA from the tissue. Reverse transcription and qPCR were employed to generate and amplify cDNA. Quality checks and size selection were performed using a Fragment Analyzer and SPRIselect reagent, and the cDNA fragments were optimized via the Illumina NextSeq Sequencing System. The final libraries, including unique indices and Illumina primers, were quantified and sequenced on the Illumina NextSeq 550 platform with paired-end sequencing. The sequencing protocol utilized 28 cycles for read 1, 10 cycles per index, and 120 cycles for read 2 on a NextSeq 500/550 High Output Kit v2.5. To enhance our understanding of the interactions that occur between immune cells and nonimmune constituents inside the tumor immune microenvironment, four patients with scRNA-seq data were subjected to paired spatial transcriptomics analysis. On the basis of our scRNA-seq results, we conducted an integrated analysis to estimate the composition of cell types and the relative proportions of different cell types at each data point in the ST analysis. To map our scRNA-seq defined cell types onto the spatial data, we utilized CytoSPACE [35], which aligns single-cell and spatial transcriptomic datasets.

### CellChat analysis

To assess the potential cell‒cell interactions between our *SPP1* + macrophage populations and other cell types, including immune cells, we employed the CellChat platform [36]. This analysis was conducted using the scRNA-seq Seurat object in R, which uses the standalone CellChat Shiny App for its Cell–Cell Communication Atlas Explorer. The cells were categorized on the basis of SingleR-defined cell type classifications, and the macrophages were further subgrouped according to their location. The analysis considered default parameterizations, and relationships involving Secreted Signaling, ECM-Receptor, and Cell‒Cell Contact were explored.

### Spatial proximity and neighborhood analysis

To quantify the spatial relationship between *SPP1* + macrophages and CD8 + T cells, we employed a neighborhood analysis approach using the Squidpy toolkit in Python [37]. After mapping cell types to spatial spots using CytoSPACE, we defined a spatial neighborhood graph based on the physical proximity of the spots. We then calculated a neighborhood enrichment score for each pair of cell types. This score represents the observed frequency of two cell types being neighbors compared to the frequency expected by random chance. A score less than 1 indicates spatial avoidance or exclusion, while a score greater than 1 suggests co-enrichment or attraction. This allowed us to quantitatively assess whether *SPP1* + macrophages and CD8 + T cells tend to be spatially segregated in the TME.

### RNA velocity analysis

To infer the developmental trajectories and cellular dynamics of macrophage subpopulations, RNA velocity analysis was conducted using the scVelo package in Python [38]. First, matrices of spliced and unspliced mRNA transcript counts were generated from the single-cell RNA sequencing data. The scVelo toolkit was then applied using the dynamical model to solve the full transcriptional dynamics and estimate the latent time and rates of gene splicing and transcription. The computed RNA velocities were subsequently projected as stream plots onto the existing UMAP embeddings to visualize the directional transitions and developmental trajectory from monocyte toward the terminally differentiated *SPP1* + macrophage state.

### RNA sequencing data processing

Tumor tissues and adjacent liver tissues were surgically resected and promptly processed. Following tissue processing, TRIzol reagent was used for RNA extraction, and the RNA concentration and integrity were assessed. Sequencing libraries were generated using the NEBNext® Ultra^TM^ RNA Library Prep Kit for Illumina®. Data analysis involved mapping reads to the human reference genome using HISAT2, with StringTie used for transcript assembly. Differential expression analysis utilized FPKM values. In proteomics data analysis, MaxQuant facilitated peptide and protein identification and quantification, followed by exploration of differential abundance and enrichment in GO terms and KEGG pathways. Gene set enrichment analysis involved annotating count data, normalization, and analyses for Gene Ontology terms and KEGG pathways.

### Stable cell lines

First, an *SPP1* gene overexpression plasmid was constructed, followed by lentiviral packaging in 293 T cells. The resulting viral particles were then transduced into the RAW264.7 cell line, and stable clones were selected through drug screening. Finally, the transfection efficiency was assessed via ELISA.

### CD8 + T cells

Following the euthanasia of OT1 mice, T cells were isolated from the spleen, and CD8 + T cells were purified using the Mouse CD8 + T Cell Isolation Kit (Stemcell, Cat#19,853) and subsequently resuspended in RPMI medium supplemented with 10% FBS, 1% sodium pyruvate, 1% nonessential amino acids, 100 U/mL penicillin‒streptomycin, 50 nM *β*-mercaptoethanol (cRPMI), and 10 ng/mL mouse IL-2. The cells were seeded at a concentration of 1 million cells/mL onto plates precoated with 5 µg/mL anti-CD3 and 2 mg/mL anti-CD28. Afterward, the cells were passaged every two days and maintained at a concentration of 1 million cells/mL. After being cultured for 2 days, these cells were used for coculture experiments.

### Animal care and ethical approval

All protocols for animal use and euthanasia were reviewed and approved by the Department of Laboratory Animal Science, Fudan University Animal Care and Ethics Committee (202307010S). Species, Strain, and Source: Female C57BL/6 mice (4 weeks old, initial weight approx. 18–20 g) were primarily used. Congenic CD45.1 and CD45.2 mice were exclusively used for the adoptive transfer study. All animals were procured from Shanghai Model Organisms Center, Inc. Housing and Welfare: Mice were housed in specific pathogen-free (SPF) conditions under a 12-h light/dark cycle with ad libitum access to food and water. Humane endpoints were strictly observed, including a body weight loss exceeding 20% or signs of severe distress. The experimental CRLM model was established in all experiments via intrasplenic injection of 5 × 10^5^ MC38 cells under anesthesia (Zoletil 100–150 μl per mouse, administered via intraperitoneal injection (i.p.)). All mice were euthanized by cervical dislocation under deep anesthesia at the experimental endpoints.

### Experimental design and procedure overview

Our study comprised two distinct and randomized animal experiments, each utilizing the single mouse as the experimental unit with a minimum sample size of six mice per group (*n* = 6). Experiment I (total *n* = 12) employed an adoptive transfer model to assess the paracrine role of macrophage *SPP1*: CD45.1 Bone Marrow-Derived Macrophages (BMDMs), which were cultured for 6 days, identified, and transiently modified (Vector or *SPP1-*sg) for 4 h, were co-injected intrasplenically with MC38 cells into CD45.2 recipient mice. This cohort was euthanized at Day 21 for analysis of tumor burden and immune cell fate tracking (CD45.1 vs. CD45.2). Experiment II (total *n* = 24) was a randomized therapeutic trial: mice inoculated with the aggressive MC38-*SPP1*-OE cells were randomized on Day 8 into four treatment arms—Isotype Control (IgG), anti-SPP1, anti-PD-1, or the combination. Antibodies were administered at 8 mg/kg via i.p. injection every 2 days for a total of 8 injections. Tumor burden and comprehensive TME immune status (via flow cytometry and mIHC) were assessed at Day 24. Notably, for experimental groups exhibiting radical tumor mass reduction (specifically the anti-SPP1 + anti-PD-1 combination group), tissues from every two treated mice were pooled prior to dissociation to ensure a sufficient number of viable single cells (> 5 × 10^5^ cells) for accurate flow cytometric antibody staining and gating analysis. No outliers or treated mice were excluded from the analysis. For all experiments, allocation was determined by computer-generated randomization, and the individuals performing necropsy, outcome measurements, and data analysis were completely blinded to the group assignments to prevent bias. No pre-established criteria for exclusion were applied, and no animals or data points were excluded from the final analyses.

### Outcome measures

The primary outcome for Experiment I was total liver tumor burden at Day 21. The primary outcome for Experiments II was the tumor surface area fraction at Day 24, calculated as the ratio of the gross tumor area to the total liver surface area using ImageJ software (v1.54p). Secondary outcomes included: detailed flow cytometric and mIHC analysis of the tumor microenvironment (TME) to quantify immune cell populations (GZMB, IFNG, TNFα, TIM3, CD8) for Experiments I and II.

### Isolation and culture of bone marrow-derived macrophages (BMDMs)

After the mice were euthanized, they were immersed in alcohol for 30 min. The skin was carefully cut, and the femur was extracted. The surrounding muscles and fascia were removed, and the femur was placed in a dish filled with PBS for washing. Both ends of the bone were scraped off to expose the marrow. A syringe was used to flush the bone cavity repeatedly with PBS to extract the bone marrow until the bone turned white. Afterward, the marrow was filtered through a strainer, and the suspension was centrifuged. The cell pellet was treated with red blood cell lysis buffer for 5 min, followed by another wash with medium. Finally, the cells were cultured in complete RPMI 1640 medium supplemented with 10% fetal bovine serum (FBS), 1% penicillin–streptomycin, and 20 ng/mL recombinant murine macrophage colony-stimulating factor (mM-CSF; PeproTech). Cells were incubated at 37 °C in a humidified atmosphere with 5% CO_2_. On Day 3, half of the culture medium was replenished with fresh M-CSF-containing complete medium [39, 40].

### Plasmids, transfection, and CRISPRi-mediated gene knockdown in primary BMDMs

For *SPP1* knockdown in primary BMDMs, we employed a CRISPRi system. Cells were seeded at 5 × 10^5^ per well in 6-well plates and cultured to 75–80% confluence before transfection. Transient transfection was carried out using Lipofectamine 3000 (Thermo Fisher Scientific, Cat# L3000015) with either the *SPP1*-KD-Cas9 plasmid, which contains a specific sgRNA targeting the *SPP1* promoter along with the dCas9 cassette, or the corresponding empty sgRNA-dCas9 vector as a negative control. Both plasmids were complexed with P3000 reagent in Opti-MEM and added dropwise to the cells. At 48 h post-transfection, culture supernatants were collected and secreted SPP1 levels were measured by ELISA (R&D Systems, Cat# MOST00). Only batches that showed a statistically significant reduction in SPP1 compared to the control group were used for subsequent functional assays and adoptive transfer experiments.

### Flow cytometry analysis of tissues

Cells were filtered and dissociated to form a single-cell suspension in DMEM or DMEM/F12. The cells were sorted via a FACSARIA III with a 100 μm nozzle in purity mode. The cell suspensions were stained with 4 μM ethidium homodimer-1 to purify the live cells. Cellular debris and cell doublets were excluded on the basis of forward and side scatter. The cells were sorted on the basis of specific markers for different populations. Single-cell suspensions from CRLM tissues were analyzed by flow cytometry. The immune cell populations were characterized using a panel of fluorochrome-conjugated antibodies. Data analysis included gating strategies to identify distinct cell subsets.

### Quantification and statistical analysis

Unless specifically noted, all the data are representative of ≥ 2 separate experiments. Experimental group assignment was determined by random designation. Statistical analyses were performed using GraphPad Prism software. The error bars represent the ± standard error of the mean (SEM) calculated using Prism. The specific statistical tests used were paired t tests, unpaired t tests, and one-way ANOVA unless otherwise noted. *P* values < 0.05 were considered to indicate statistical significance. Investigators were not blinded to group assignment during experimental procedures or analysis.

For all genome-wide analyses including single-cell differential expression, Gene Set Enrichment Analysis (GSEA), and Gene Set Variation Analysis (GSVA), *p*-values were adjusted for multiple comparisons using the Benjamini-Hochberg (BH) procedure to control the False Discovery Rate (FDR). An adjusted *p*-value (or *q*-value) of < 0.05 was defined as the threshold for statistical significance unless otherwise noted. In all figures, statistical significance is denoted by asterisks (*), where * indicates* p* < 0.05, ** indicates *p* < 0.01, *** indicates *p* < 0.001, and **** indicates *p* < 0.0001. For all comparisons, the exact *P*-values or FDR values are provided within the corresponding Figure Legends to ensure full transparency and reproducibility.

## Supplementary Information

Supplementary Material 1.

## Data Availability

The raw single-cell RNA sequencing (scRNA-seq) and spatial transcriptomics datasets generated in this study have been deposited in the National Genomics Data Center (NGDC), part of the China National Center for Bioinformation (CNCB), under the umbrella accession number PRJCA067146 (https://ngdc.cncb.ac.cn/bioproject/browse/PRJCA067146). The processed data matrices supporting the primary findings are available from the corresponding author upon reasonable request. Additionally, publicly available datasets utilized in this study were retrieved from the NCBI Gene Expression Omnibus (GEO) database under the accession numbers GSE13294, GSE14333, GSE17538, and GSE26682. All other remaining raw data that support the conclusions of this article are available from the corresponding author upon request.
