# Supplementary material for: Characterization of SPP1 + macrophages and their correlation with resistance to neoadjuvant chemotherapy in colorectal cancer liver metastasis
Source: Mol Biomed. 2026 Oct 5;7:194. doi: 10.1186/s43556-026-00581-7 (PMC13635084; doi:10.1186/s43556-026-00581-7)
Supplement: Supplementary file 1 [file 43556_2026_581_MOESM1_ESM.pdf]

Characterization of *SPP1*+ Macrophages and Their Correlation with Resistance to Neoadjuvant Chemotherapy in Colorectal Cancer Liver Metastasis.

Jingzhi Tang[1,2,†], **Weijie Hao**[3,†], **Xigan He**[4,†], Xin Qian[5], Linjie Ruan[6], Mengyao Quan[1,2], Jian Zhou[1,2], Jing Tang[7], Lu Wang[4], Yi Guo[8,*], Yiming Zhao[4,*], Cai Chang[1,2,*], Shichong Zhou[1,2,*].

Affiliations:

1, Department of Ultrasonography, Fudan University Shanghai Cancer Center, Shanghai, 200032, China

2, Department of Oncology, Shanghai Medical College, Fudan University, Shanghai,200032, China

3, State Key Laboratory of Oncogenes and Related Genes, Renji-Med X Clinical Stem Cell Research Center, Ren Ji Hospital, School of Medicine, Shanghai Jiao Tong University, Shanghai 200032, China

4, Department of Hepatic Surgery, Fudan University Shanghai Cancer Center, Shanghai 200032, China

5, Department of Vascular Biology, European Center for Angioscience (ECAS), Medical Faculty Mannheim, Heidelberg University, 68167, Mannheim, Germany

6, Department of Integrative Oncology, Fudan University Shanghai Cancer Center, Shanghai 200032, China

7, Research Program in Systems Oncology, Faculty of Medicine, University of Helsinki, Helsinki, Finland, 00290

8, Department of Electronic Engineering, School of Information Science and Technology, Fudan University, Shanghai 200433, China; The Key Laboratory of Medical Imaging Computing and Computer Assisted Intervention (MICCAI) of Shanghai, Shanghai 200032, China

† These authors contributed equally

* Correspondence: guoyi@fudan.edu.cn (Y.G.), gomas1711@163.com (Y.Z.), changc61@163.com (C.C.), sczhou@fudan.edu.cn (S.Z.)

ORCID: 0000-0001-5849-2368

Keywords: Colorectal cancer liver metastasis; Neoadjuvant chemotherapy resistance; spatial immune barrier; *SPP1*+ macrophages; CD8+ T cell exhaustion

Fig. S1

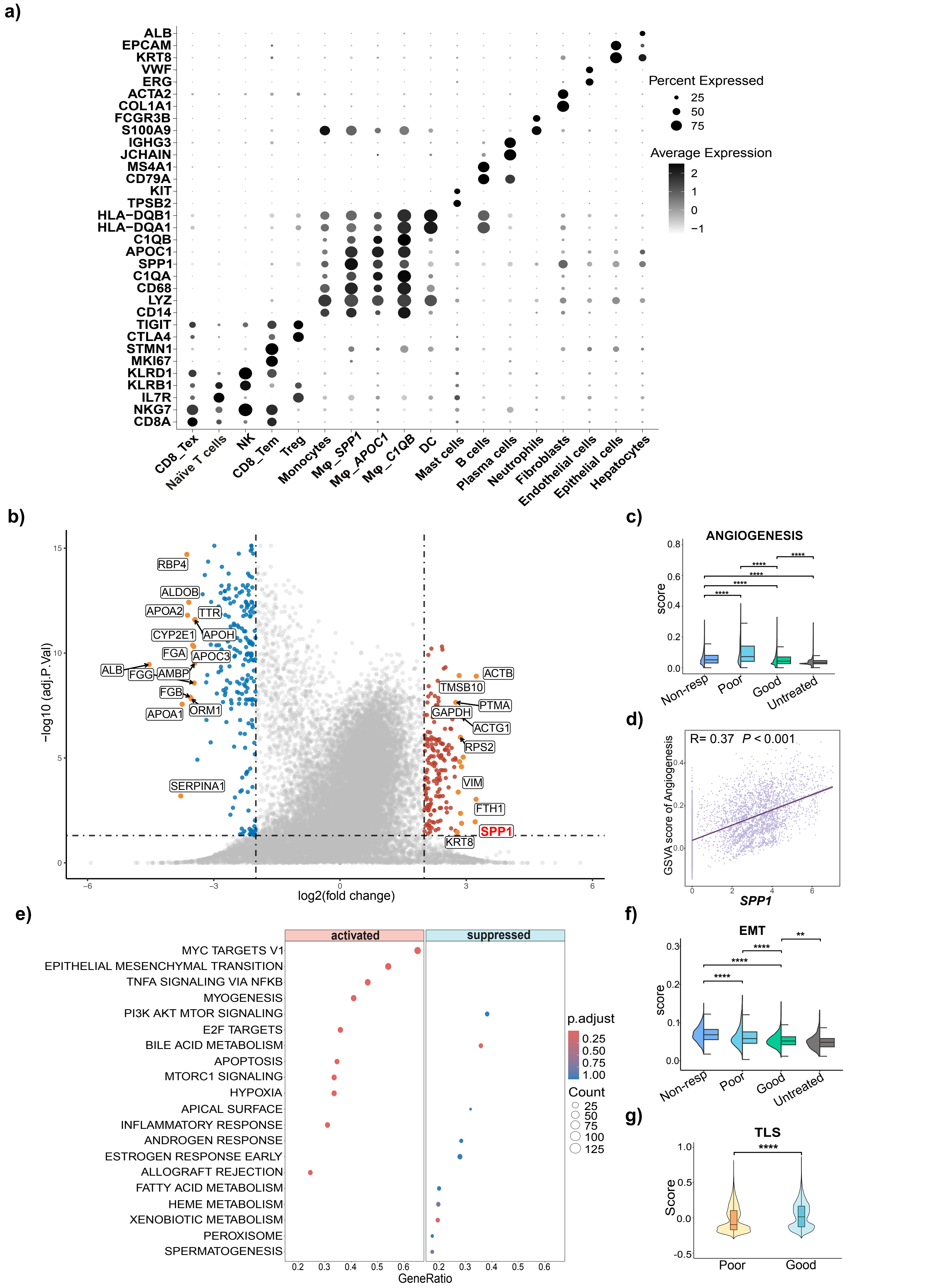
a)
ALB
EPCAM
KRT8
VWF
ERG
ACTA2
COL1A1
FCGR3B
S100A9
IGHG3
JCHAIN
MS4A1
CD79A
KIT
TPSB2
HLA−DQB1
HLA−DQA1
C1QB
APOC1
SPP1
C1QA
CD68
LYZ
CD14
TIGIT
CTLA4
STMN1
MKI67
KLRD1
KLRB1
IL7R
NKG7
CD8A
CD8_Tex
Naïve T cells
NK
CD8_Tem
Treg
Monocytes
Mφ_SPP1
Mφ_APOC1
Mφ_C1QB
DC
Mast cells
B cells
Plasma cells
Neutrophils
Fibroblasts
Endothelial cells
Epithelial cells
Hepatocytes
Percent Expressed
25
50
75
Average Expression
2
1
0
−1
b)
RBP4
ALDOB
APOA2
TTR
CYP2E1
APOH
FGA
APOC3
ALB
FGG
AMBP
FGB
ORM1
APOA1
SERPINA1
ACTB
TMSB10
PTMA
GAPDH
ACTG1
RPS2
VIM
FTH1
SPP1
KRT8
−log10 (adj.P.Val)
log2(fold change)
c)
ANGIOGENESIS
score
Non-resp
Poor
Good
Untreated
d)
R= 0.37 P < 0.001
GSVA score of Angiogenesis
SPP1
e)
activated
suppressed
MYC TARGETS V1
EPITHELIAL MESENCHYMAL TRANSITION
TNFA SIGNALING VIA NFKB
MYOGENESIS
PI3K AKT MTOR SIGNALING
E2F TARGETS
BILE ACID METABOLISM
APOPTOSIS
MTORC1 SIGNALING
HYPOXIA
APICAL SURFACE
INFLAMMATORY RESPONSE
ANDROGEN RESPONSE
ESTROGEN RESPONSE EARLY
ALLOGRAFT REJECTION
FATTY ACID METABOLISM
HEME METABOLISM
XENOBIOTIC METABOLISM
PEROXISOME
SPERMATOGENESIS
p.adjust
0.25
0.50
0.75
1.00
Count
25
50
75
100
125
GeneRatio
f)
EMT
score
Non-resp
Poor
Good
Untreated
g)
TLS
Score
Poor
Good

Fig. S2

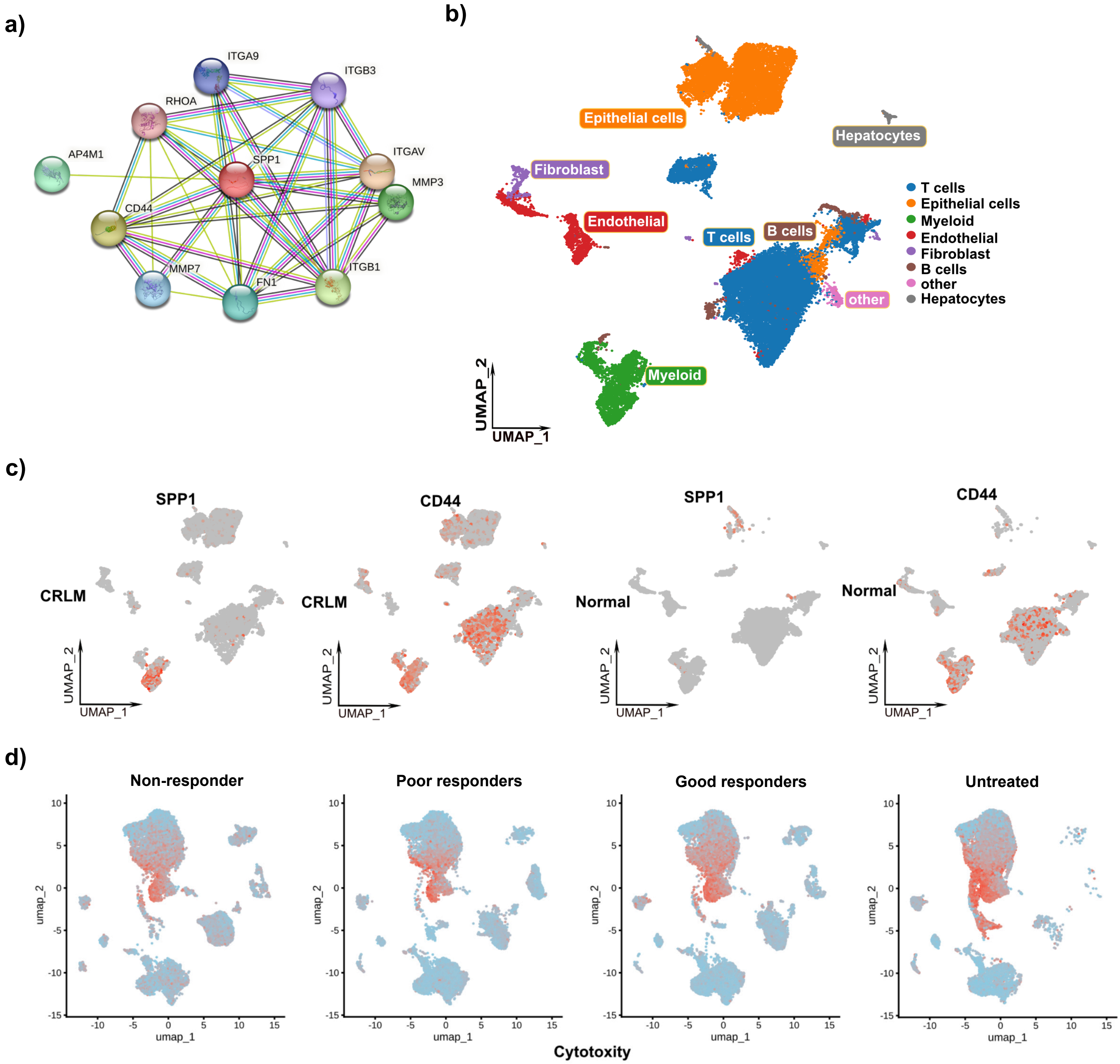

a)
ITGA9
ITGB3
RHOA
AP4M1
SPP1
ITGAV
MMP3
CD44
MMP7
FN1
ITGB1
b)
Epithelial cells
Hepatocytes
Fibroblast
Endothelial
T cells
B cells
other
Myeloid
T cells
Epithelial cells
Myeloid
Endothelial
Fibroblast
B cells
other
Hepatocytes
UMAP_2
UMAP_1
c)
SPP1
CD44
SPP1
CD44
CRLM
CRLM
Normal
Normal
UMAP_2
UMAP_1
d)
Non-responder
Poor responders
Good responders
Untreated
umap_2
umap_1
Cytotoxity

Fig. S3

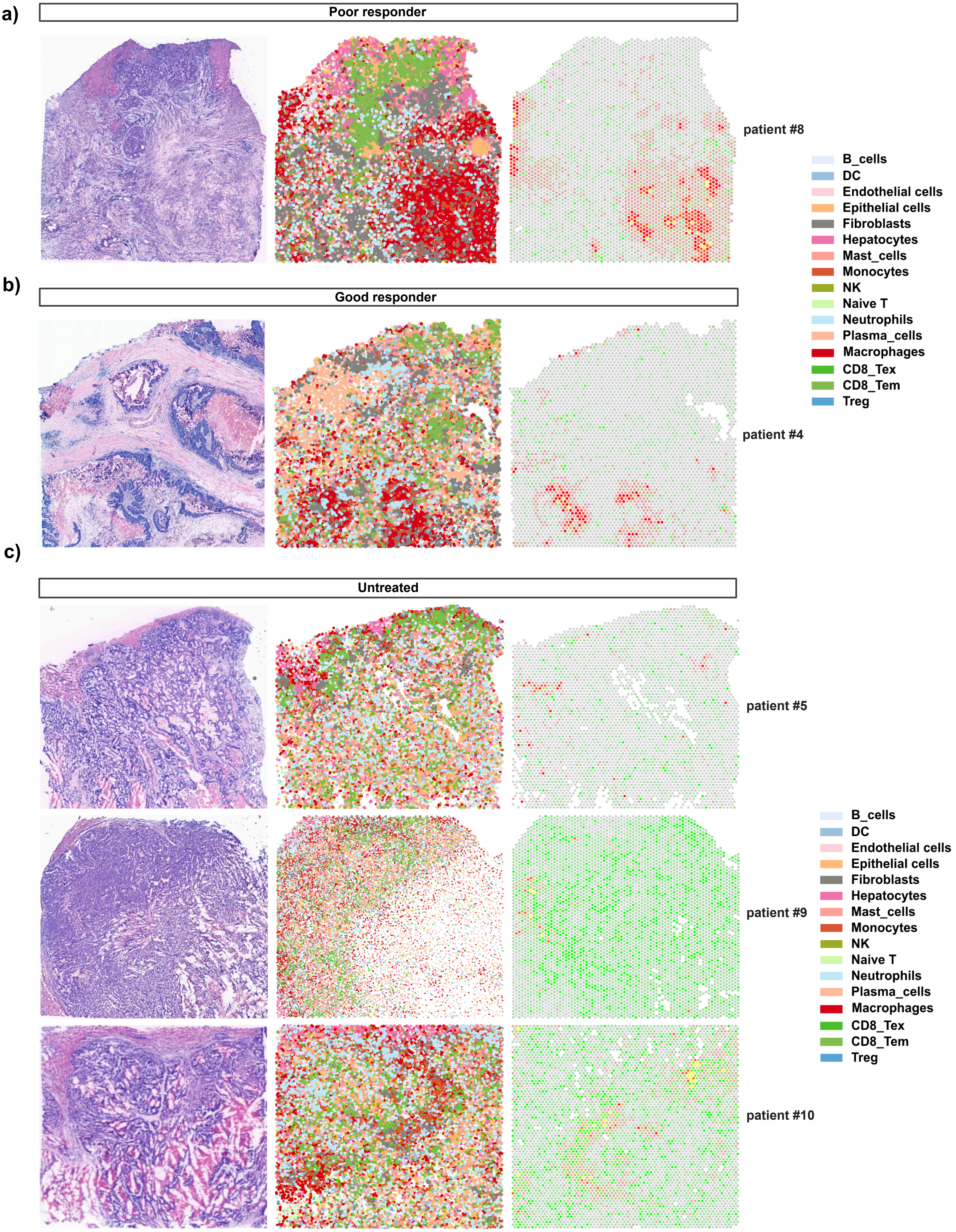

a)
Poor responder
patient #8
b)
Good responder
patient #4
c)
Untreated
patient #5
patient #9
patient #10
B_cells
DC
Endothelial cells
Epithelial cells
Fibroblasts
Hepatocytes
Mast_cells
Monocytes
NK
Naive T
Neutrophils
Plasma_cells
Macrophages
CD8_Tex
CD8_Tem
Treg

Fig. S4

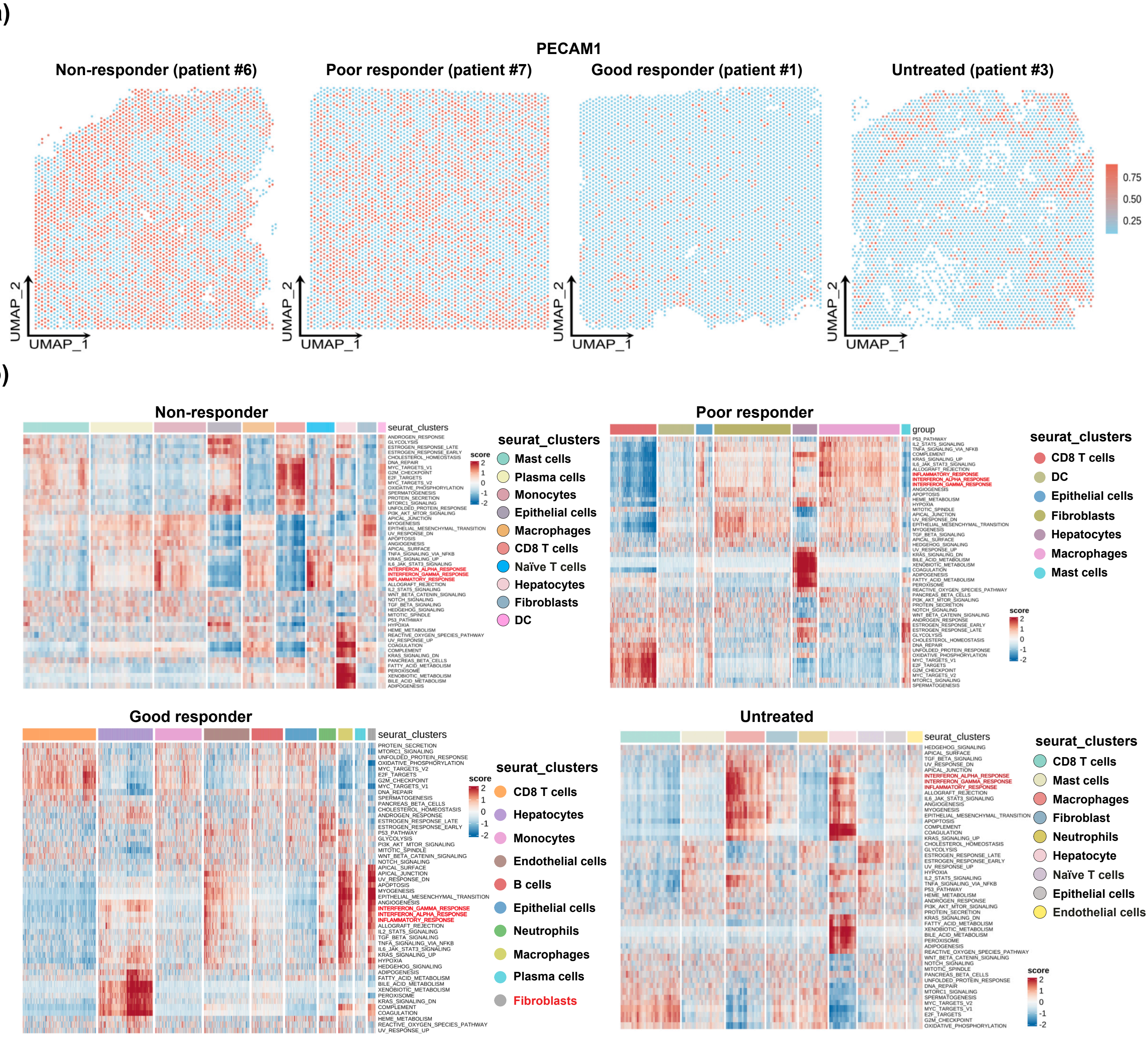
a)
PECAM1
Non-responder (patient #6)
Poor responder (patient #7)
Good responder (patient #1)
Untreated (patient #3)
UMAP_2
UMAP_1
0.75
0.50
0.25
b)
Non-responder
seurat_clusters
Mast cells
Plasma cells
Monocytes
Epithelial cells
Macrophages
CD8 T cells
Naïve T cells
Hepatocytes
Fibroblasts
DC
Poor responder
group
seurat_clusters
CD8 T cells
DC
Epithelial cells
Fibroblasts
Hepatocytes
Macrophages
Mast cells
Good responder
seurat_clusters
CD8 T cells
Hepatocytes
Monocytes
Endothelial cells
B cells
Epithelial cells
Neutrophils
Macrophages
Plasma cells
Fibroblasts
Untreated
seurat_clusters
CD8 T cells
Mast cells
Macrophages
Fibroblast
Neutrophils
Hepatocyte
Naïve T cells
Epithelial cells
Endothelial cells
score
2
1
0
-1
-2

Table S1. Clinical characteristics of patients included in this study

1. Clinical characteristics of patients of cohort (scRNA-seq).

Patient_ID	Neoadjuvant chemotherapy	TNM	NAC treatment	Pathological classification	Method
P1	poor responder	T2N0M1	XELOX	Adenocarcinoma	scRNA-seq
P2	good responder	T3N1M1	XELOX	Adenocarcinoma	scRNA-seq
P3	non-responder	T3N1M1	XELOX	Adenocarcinoma	scRNA-seq
P4	good responder	T3N1M1	FOLFIRI	Adenocarcinoma	scRNA-seq
P5	poor responder	T3N1M1	XELOX	Adenocarcinoma	scRNA-seq
P6	untreated	T3N1M1	No	Adenocarcinoma	scRNA-seq
P7	untreated	T3N1M1	No	Adenocarcinoma	scRNA-seq
P8	good responder	T3N0M1	FOLFIRI	Adenocarcinoma	scRNA-seq
P9	good responder	T2N0M1	XELOX	Adenocarcinoma	scRNA-seq
P10	poor responder	T2N0M1	XELOX	Adenocarcinoma	scRNA-seq

2. Clinical characteristics of patients of cohort (ST).

Patient_ID	Neoadjuvant chemotherapy	TNM	NAC treatment	Pathological classification	Method
P1	good responder	T3N1M1	FOLFOX	Adenocarcinoma	ST
P3	untreated	T3N1M1	No	Adenocarcinoma	ST
P4	good responder	T2N0M1	FOLFOX	Adenocarcinoma	ST
P5	untreated	T3N1M1	No	Adenocarcinoma	ST
P6	non-responder	T2N0M1	FOLFOX	Adenocarcinoma	ST
P7	poor responder	T2N0M1	XELOX	Adenocarcinoma	ST
P8	poor responder	T3N1M1	XELOX	Adenocarcinoma	ST
P9	untreated	T3N1M1	No	Adenocarcinoma	ST
P10	untreated	T3N1M1	No	Adenocarcinoma	ST

Table S2. The antibodies and reagent involved in this article.

Antibodies and reagents	Supplier	Cat no.
AF488 anti-human CD45	BioLegend	304017
APC/Cy7 anti-human CD3	BioLegend	344840
PE/Cy7 anti-human CD4	BioLegend	300511
APC anti-human CD8a	BioLegend	300911
FITC anti-mouse CD45	BioLegend	157214
PE anti-mouse CD3	BioLegend	100206
BV605 anti-mouse CD4	BioLegend	100547
PerCP/Cy5.5 anti-mouse CD8a	BioLegend	100734
Pharmingen™ APC Mouse Anti-Human CD25	Biosciences	567317
anti-mouse GZMB	BioLegend	396406
anti-mouse IFNG	BioLegend	505808
anti-mouse TNFa	BioLegend	506308
anti-mouse TIM3	abcam	ab241332
anti-human SPP1	abcam	ab214050
anti-human CD44	CST	37259
anti-human COL1A1	CST	72026
anti-human PD1	abcam	ab52587
anti-human CD8	abcam	ab245118
anti-human CD68	abcam	ab213363
anti-mouse CD8	abcam	ab217344
Alexa Fluor® 488 anti-mouse CD45.2	BioLegend	109816
Ultra-LEAF™ Purified anti-mouse CD28 Ant ibody	BioLegend	102121
Ultra-LEAF™ Purified anti-mouse CD3	BioLegend	100253
Precision Count Beads™	BioLegend	424902
Mouse CD8+ TCell Isolation Kit	Stemcell	19853
eBioscience^{Tm}Foxp3/Transcription Factor Staining Buffer Set	Invitrogen	00-5523-00
Zombie Aqua™ Fixable Viability Kit	Biolegend	423102
InVivoMAb anti-mouse osteopontin	Bioxcell	BE0372
InVivoMAb mouse IgG1 isotype control	Bioxcell	BE0083
InVivoMAb anti-mouse PD-1(CD279)	Bioxcell	BE0146

Supplementary Figure Legends

Supplementary Figure 1: scRNA-seq Data Characterization and Functional Annotation of *SPP1*+ Macrophages.

(a) Dot plot showing the expression of canonical marker genes used to annotate the 18 cell clusters identified in Fig. 1b.

(b) Volcano map of differential genes between liver metastasis sites and paired adjacent liver tissues in patients with CRLM. The red dots represent genes upregulated in liver metastasis sites and the blue dots represent genes upregulated in paired adjacent liver tissues.

(c) Violin plots of GSVA scores for Angiogenesis pathways, compared between patient groups.

(d) Scatter plot showing the positive correlation between *SPP1* gene expression and the GSVA scores for Angiogenesis pathways across all single cells. R and *P* values from Pearson correlation are shown.

(e) GSEA plots showing enrichment of pro-tumoral pathways (Epithelial Mesenchymal Transition, MYC_TARGETS_V1) in *SPP1*+ macrophages from poor responders compared to good responders.

(f-g) Violin plots of GSVA scores for EMT, and Tertiary Lymphoid Structures (TLS) pathways, compared between patient groups.

Statistical significance in (d) was determined by one-way ANOVA. **$p < 0.01$, ****$p < 0.0001$.

Supplementary Figure 2: Regulatory Network and Independent Cohort Validation.

(a) Protein-protein interaction (PPI) network of SPP1 and its closely associated partners, constructed using the STRING database.

(b) UMAP of scRNA-seq transcriptome profiles in CRLM patients. Data wer obtained from Anuja Sathe et al.

(c) UMAP annotated with the expression of *SPP1* and *CD44* in CRLM and paired adjacent liver tissue (normal).

(d) The immune-related score of cytotoxity among CRLM. Pathway is predominantly distributed in CD8+ T cells and NK.

Supplementary Figure 3: Additional Spatial Transcriptomics Data.

(a-c) Additional representative ST data from patients in the poor responder, good responder, and untreated groups, showing H&E staining and corresponding CytoSPACE cell-type deconvolution maps.

Supplementary Figure 4: *SPP1*+ macrophages contributes to an attenuated anti-tumour immune response.

(a) Visualization of the angiogenesis marker *PECAM1* expression on representative ST slides from each patient group.

(b) The GSVA score of different type of cell clusters in CRLM patient.
